# Predicting control of cardiovascular disease risk factors in South Asia using machine learning

**DOI:** 10.1038/s41746-024-01353-9

**Published:** 2024-12-10

**Authors:** Anna Reuter, Mohammed K. Ali, Viswanathan Mohan, Lydia Chwastiak, Kavita Singh, K. M. Venkat Narayan, Dorairaj Prabhakaran, Nikhil Tandon, Nikkil Sudharsanan

**Affiliations:** 1 German Federal Institute of Population Research, Wiesbaden, Germany; 2 Heidelberg Institute of Global Health, Heidelberg University, Heidelberg, Germany; 3 Emory Global Diabetes Research Center, Woodruff Health Sciences Center and Emory University, Atlanta, GA USA; 4 Dr. Mohan’s Diabetes Specialties Centre, Chennai, India; 5 Diabetology, Madras Diabetes Research Foundation, Chennai, India; 6 Department of Psychiatry and Behavioral Sciences, University of Washington, Seattle, WA USA; 7 Centre for Control of Chronic Conditions, Public Health Foundation of India, Gurgaon, India; 8 Department of Endocrinology and Metabolism, All India Institute of Medical Sciences, New Delhi, India; 9 TUM School of Medicine and Health, Technical University of Munich, Munich, Germany; 10 Munich Center for Health Economics and Policy, Munich, Germany

**Keywords:** Health care, Cardiovascular diseases, Risk factors

## Abstract

A substantial share of patients at risk of developing cardiovascular disease (CVD) fail to achieve control of CVD risk factors, but clinicians lack a structured approach to identify these patients. We applied machine learning to longitudinal data from two completed randomized controlled trials among 1502 individuals with diabetes in urban India and Pakistan. Using commonly available clinical data, we predict each individual’s risk of failing to achieve CVD risk factor control goals or meaningful improvements in risk factors at one year after baseline. When classifying those in the top quartile of predicted risk scores as at risk of failing to achieve goals or meaningful improvements, the precision for not achieving goals was 73% for HbA1c, 30% for SBP, and 24% for LDL, and for not achieving meaningful improvements 88% for HbA1c, 87% for SBP, and 85% for LDL. Such models could be integrated into routine care and enable efficient and targeted delivery of health resources in resource-constrained settings.

## Introduction

Over the past decades, the burden of cardiovascular diseases (CVD) has been rising in South Asia, both in terms of mortality and disability-adjusted life years^1,2^. The risk of CVD can be substantially reduced by controlling blood glucose, blood pressure, and lipids^3,4^, but patients repeatedly fail to achieve these goals. For example, a recent study using nationally representative data from India showed that only 36%, 49%, and 42% of individuals receiving care for type 2 diabetes achieved blood glucose control (HbA1c < 7%), blood pressure control (<140/90 mmHg), and LDL cholesterol control (<100 mg/dL) respectively^5^.

Additional healthcare interventions may help individuals achieve their care goals. For example, past studies showed that complementary care management by nurses, lay people, or peers, as well as the use of mobile phones to provide reminders or advice, can help to improve the management of CVD risk factors among individuals with diabetes^6–8^. But even if such interventions are cost-effective in the long-run, they might not be feasible to implement for entire patient populations due to lack of staff, infrastructure, and financial resources^9^. Available resources may need to be targeted to the patients most in need of additional assistance. Yet it is unclear how to identify such individuals for intervention targeting.

We developed machine learning algorithms which rank and categorize individuals based on their predicted risk of failing to achieve CVD risk factor control, or failing to achieve meaningful reductions in CVD risk factor levels. The resulting models are similar to widely-used CVD risk scores such as the ACC/AHA ASCVD Risk Score^10^, but with the goal to identify the patient’s risk of not meeting CVD care goals rather than their risk of a CVD endpoint. We took advantage of rare longitudinal clinical data collected with high quality protocols on diabetic patients in India and Pakistan, the CARRS and the INDEPENDENT trials^11–13^. We built our machine learning models from commonly available characteristics, such as biomarkers routinely collected in CVD care, medical history, and basic sociodemographic characteristics. This enables the use of the models in real clinical settings. Our resulting models can thus be provided as a calculator for clinicians and health administers, allowing them to easily determine how to target additional efforts and improve CVD risk factor control among their patients.

## Results

### Sample characteristics

Women comprised 60% of the INDEPENDENT sample and 54% of the CARRS sample (Table 1). In both samples, patients were about 53 years old on average. Patients in the CARRS sample had poorer CVD risk factor levels at baseline compared to patients from the INDEPENDENT sample. After 12 months, in the INDEPENDENT sample, the share of patients with an HbA1 ≥ 8% (“failure to achieve HbA1c control”) reduced from 71% to 50%, the share of patients with SBP ≥ 140 mmHg (“failure to achieve SBP control”) from 43% to 18%, and the share of patients with LDL ≥ 130 mg/dL (“failure to achieve LDL control”) from 25% to 15%. In the CARRS data, the share of patients with HbA1 ≥ 8% reduced from 100% to 60%, the share of patients with SBP ≥ 140 mmHg from 69% to 25%, and the share of patients with LDL ≥ 130 mg/dL from 48% to 21%. In the INDEPENDENT sample, 60% of the patients had a reduction in HbA1c of less than 1 percentage point (“failure to achieve clinically meaningful reduction in HbA1c”), 60% a reduction of less than 10 mmHg in SBP (“failure to achieve clinically meaningful reduction in SBP”), and 58% a reduction of less than 10 mg/dL in LDL (“failure to achieve clinically meaningful reduction in LDL”). In the CARRS sample, 46% did not achieve a clinically meaningful reduction in HbA1c, 44% in SBP, and 44% in LDL levels.

**Table 1:** Sample characteristics at baseline and clinical outcomes after 12 months

	CARRS (1115 patients)		INDEPENDENT (387 patients)	
	Mean	sd	Mean	sd
Demographics				
Female, %	54.1		59.7	
Age, years	53.6	9.2	52.5	8.5
Education, %				
Professional	7.1		9.0	
Graduate	19.7		14.0	
Secondary	43.7		50.9	
Primary	11.7		16.8	
Literate	5.6		2.3	
Illiterate	12.3		7.0	
Occupation, %				
Professional	6.4		7.2	
Trained	15.3		9.6	
Skilled	7.9		12.1	
Semi-skilled	5.0		7.2	
Unskilled	4.8		4.4	
Homemaker	40.8		50.4	
Retired	14.0		8.0	
Unemployed	4.1		1.0	
Other	1.6		0.0	
Biomarkers				
HbA1c, percentage points	9.9	1.6	9.1	1.9
Systolic blood pressure, mmHg	143.3	19.5	132.3	15.9
Diastolic blood pressure, mmHg	81.7	10.9	80.3	9.9
Total cholesterol, mg/dl	195.4	44.8	173.5	43.7
HDL, mg/dl	44.1	8.9	41.8	11.5
LDL, mg/dl	122.6	37.1	101.5	37.5
Outcomes at baseline				
HbA1c ≥ 8%, %	100.0		71.1	
SBP ≥ 140 mmHg, %	69.3		42.9	
LDL ≥ 130 mg/dL, %	47.6		24.6	
Outcomes after 12 months				
HbA1c ≥ 8%, %	60.4		50.1	
SBP ≥ 140 mmHg, %	25.0		18.3	
LDL ≥ 130 mg/dL, %	21.0		14.7	
HbA1c reduction < 1 percentage point, %	46.4		60.0	
SBP reduction < 10 mmHg, %	44.2		59.8	
LDL reduction < 10 mg/dL, %	43.6		57.8	

Due to missing information on specific outcomes after 12 months for about 7% patients (HbA1c: 103, SBP: 101, LDL: 108), the sample size slightly differs for these outcomes.

### Selected models and composition

**Fig. 1:**
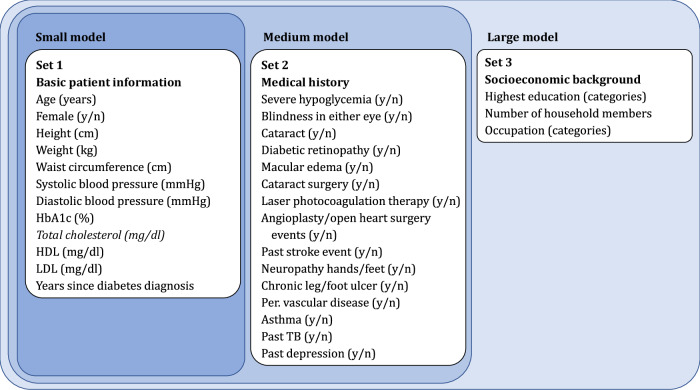
Predictors used in the small, medium, and large machine learning models. Predictors come from baseline data of the CARRS and INDEPENDENT trials. Units respectively scales in brackets. Total cholesterol was omitted after the preselection based on its high correlation (>0.8) with other predictors.

We ran boosted logistic models, boosted tree models, and support vector machines (SVM) on three different sets of predictors, shown in Fig. 1. We developed our models based on a random 80% subset of the CARRS data, tested on the remaining 20%, and validated on the INDEPENDENT data. The 10-fold cross-validation resulted in the selection of boosted logistic models for all outcomes except failure to achieve SBP improvements with the medium-sized (basic patient information and medical history) model for predicting failure to achieve HbA1c control, LDL control, and LDL improvement, the small (basic patient information only) model for failure to achieve SBP control, and the large model (all predictors) for not achieving meaningful HbA1c improvements (parameter specifications shown in Supplementary Table 2). For failure to achieve SBP improvement, the small boosted tree model was chosen. While the larger models were selected based on their higher median AUC, the small models using only basic patient information performed similarly to larger models (Supplementary Fig. 1, Supplementary Tables 3–5).

### Variable importance

**Fig. 2:**
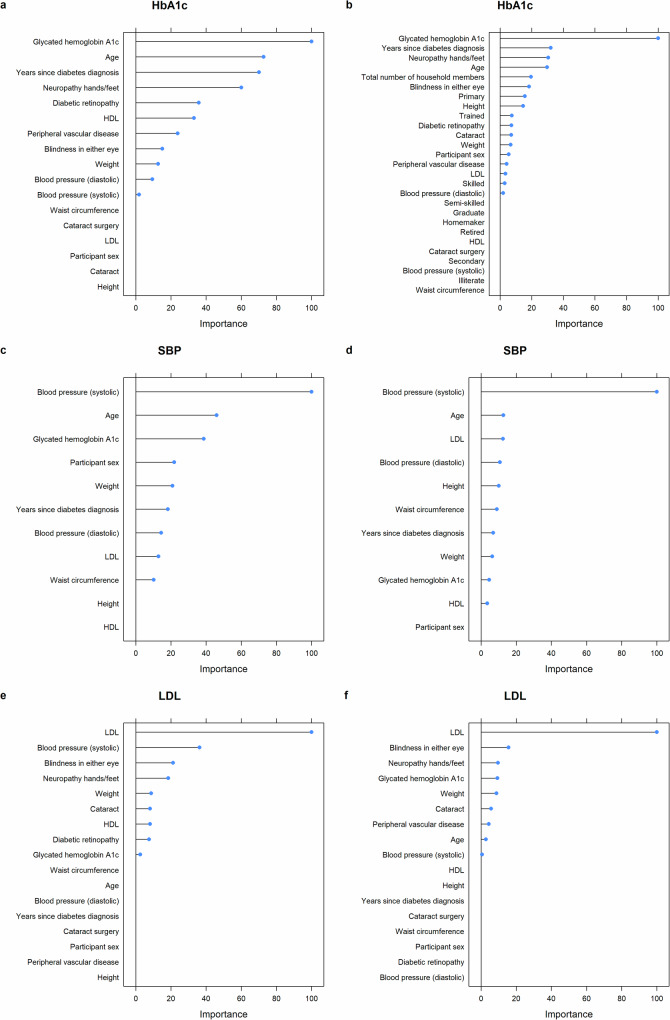
Variable importance plots. **a** Not achieving HbA1c control. **b** Not achieving HbA1c improvement. **c** Not achieving SBP control. **d** Not achieving SBP improvement. **e** Not achieving LDL control. **f** Not achieving LDL improvement.

For all selected models, the baseline measurement of the respective risk factor (e.g., baseline HbA1c levels for failure to achieve HbA1c control) is the most important predictor (Fig. 2). For HbA1c, the years since the diabetes diagnosis, age, and neuropathy in hands or feet are important predictors of failure to achieve control and meaningful reductions in HbA1c. For not achieving SBP control, age and HbA1c are important predictors, while for not achieving SBP improvements, basic patient information plays a comparatively small role. For both LDL outcomes, blindness and neuropathy in hands or feed are among the top predictors, though with a relatively low variable importance score. For not achieving LDL control, SBP is among the top predictors as well, while for not achieving LDL improvements, HbA1c is in the top ranking.

### Risk score

**Fig. 3:**
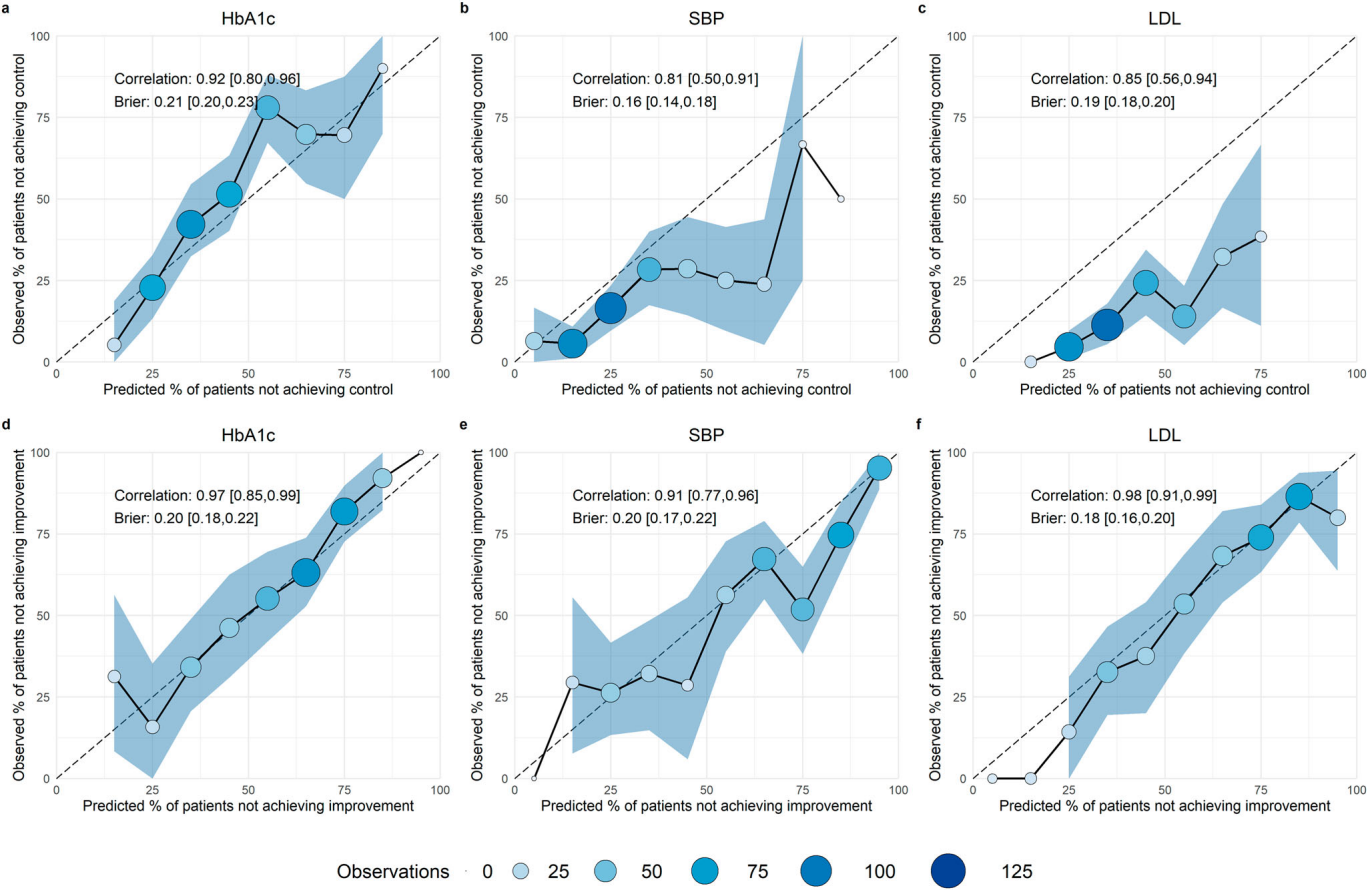
Calibration curves. The selected models in Panels (**a**-**c**) are the medium-sized boosted logistic model for HbA1c, the small boosted logistic model for SBP, and the medium-sized boosted logistic model for LDL. The selected models in Panels **d**–**f** are the large boosted logistic model for HbA1c, the small boosted tree model for SBP, and the medium-sized boosted logistic model for LDL. Point size and fill intensity reflect the relative sample size of the respective ten percentage point bin, with larger and darker points corresponding to a larger share of patients included in this bin, and empty bins not shown. Shaded areas represent the confidence intervals, obtained from bootstrapping with 1000 draws, where they could be calculated. **a** Not achieving HbA1c control. **b** Not achieving SBP control. **c** Not achieving LDL control. **d** Not achieving HbA1c improvement. **e** Not achieving SBP improvement. **f** Not achieving LDL improvement.

In Panels a–c of Fig. 3, we graphed the model-based predicted risk that an individual will not meet a CVD goal against the actual proportion of individuals that did not meet the goal in the validation data (the INDEPENDENT sample). Across models and outcomes, the predicted risk was highly correlated with the observed risk (HbA1c: 0.92, SBP: 0.81, LDL: 0.85). There were stronger correlations between the predicted and observed risk for meaningful improvement outcomes (Panels d–f; HbA1c: 0.97, SBP: 0.91, LDL: 0.98). The Brier score varied around 0.2 for all outcomes except SBP control (HbA1c control: 0.21, SBP control: 0.16, LDL control: 0.19, HbA1c improvement: 0.20, SBP improvement: 0.20, LDL improvement: 0.18), with a Brier score of 0 indicating perfect prediction and a Brier score of 1 the worst prediction possible. The models predicted only low to medium risk of failure to achieve SBP and LDL control for the patients in the validation set. Still, the models overestimated the risk of failure to achieve control for both outcomes. This is similar to the prediction on the testing set, i.e., the remaining 20% of the CARRS sample (Supplementary Note 3 and Supplementary Fig. 2). For the time-shifted data (applying the models on predictors after 12 months to predict outcomes after 24 months), the models underestimated the risk for failure to achieve HbA1c control and improvement, but overestimated the risk of failure to achieve control (Supplementary Note 4 and Supplementary Fig. 4).

### Targeting based on ranking

Table 2 presents the precision and sensitivity in the validation data when classifying individuals as at risk depending on different percentiles of the predicted risk score (a graphical representation over all percentiles can be found in Supplementary Note 3 and Supplementary Fig. 3). When classifying those in the top 10% of the predicted risk score as at risk, the actual prevalence of risk factor control failure among those classified as at risk (precision) was 73% [95% confidence interval: 59%,89%] for HbA1c, 35% [19%,51%] for SBP, and 35% [19%,49%] for LDL. This cutoff results in a sensitivity of 14% [12%,17%] for HbA1c, 19% [11%,26%] for SBP, and 24% [13%,32%] for LDL. When classifying those in the top 25% of the predicted risk score as at risk, the precision was 73% [64%,82%] for HbA1c, 30% [20%,40%] for SBP, and 24% [15%,32%] for LDL. At this cutoff, the sensitivity was 37% [32%,41%] for HbA1c, 41% [30%,52%] for SBP, and 40% [28%, 52%] for LDL. When classifying those in the top 50% of the predicted risk score as at risk, the precision was 68% [61%,75%] for HbA1c, 28% [22%,35%] for SBP, and 21% [16%,28%] for LDL. This cutoff resulted in a sensitivity of 67% [62%,73%] for HbA1c, 75% [68%,86%] for SBP, and 73% [62%,84%] for LDL.

**Table 2:** Performance of the machine learning predictions when patients are targeted according to top risk percentiles

Outcome definition	Not achieving control			Not achieving improvements		
CVD risk factor	HbA1c	SBP	LDL	HbA1c	SBP	LDL
Chosen specification	Logistic (M)	Logistic (S)	Logistic (M)	Logistic (L)	Tree (S)	Logistic (M)
Observations	377	378	374	377	378	374
Number of positives	189	69	55	226	226	216
Prevalence	50%	18%	15%	60%	60%	58%
[95% CI]	[45%,56%]	[14%,22%]	[11%,18%]	[55%, 65%]	[55%, 65%]	[53%,63%]
Targeted percentile	Top 10%					
Precision	73%	35%	35%	92%	92%	84%
[95% CI]	[59%,89%]	[19%,51%]	[19%,49%]	[81%,100%]	[81%,100%]	[69%,95%]
Sensitivity	14%	19%	24%	15%	15%	14%
[95% CI]	[12%,17%]	[11%,26%]	[13%,32%]	[13%, 17%]	[13%, 17%]	[12%,16%]
Targeted percentile	Top 25%					
Precision	73%	30%	24%	88%	87%	85%
[95% CI]	[64%,82%]	[20%,40%]	[15%,32%]	[81%, 95%]	[80%, 95%]	[77%,91%]
Sensitivity	37%	41%	40%	37%	36%	37%
[95% CI]	[32%,41%]	[30%,52%]	[28%,52%]	[33%, 40%]	[33%, 39%]	[33%,40%]
Targeted percentile	Top 50%					
Precision	68%	28%	21%	77%	75%	79%
[95% CI]	[61%,75%]	[22%,35%]	[16%,28%]	[70%, 83%]	[69%, 82%]	[73%,85%]
Sensitivity	67%	75%	73%	64%	62%	69%
[95% CI]	[62%,73%]	[68%,86%]	[62%,84%]	[59%, 68%]	[58%, 67%]	[64%,74%]

This is the performance of the models with the highest median area under the curve over all cross-validation folds applied to the validation data. Number of positives is the number of patients not achieving the outcome. Precision is the percentage of selected patients not achieving the outcome. Sensitivity is the percentage of patients not achieving the outcome who are selected. Confidence intervals obtained from bootstrapping with 1000 draws.

For failure to achieve meaningful risk factor reductions, when classifying those in the top 10% of the predicted risk score as at risk, the precision was 92% [81%,100%] for HbA1c, 92% [81%,100%] for SBP, and 84% [69%,95%] for LDL. At this cutoff, the sensitivity was 15% [13%,17%] for HbA1c, 15% [13%,17%] for SBP, and 14% [12%,16%] for LDL. When classifying those in the top 25% of the predicted risk score as at risk, the precision was 88% [81%,95%] for HbA1c, 87% [80%,95%] for SBP, and 85% [77%,91%] for LDL. This cutoff resulted in a sensitivity of 37% [33%,40%] for HbA1c, 36% [33%,39%] for SBP, and 37% [33%,40%] for LDL. When classifying those in the top 50% of the predicted risk score as at risk, the precision was 77% [70%,83%] for HbA1c, 75% [69%,82%] for SBP, and 79% [73%,85%] for LDL. This cutoff resulted in a sensitivity of 64% [59%,68%] for HbA1c, 62% [58%,67%] for SBP, and 69% [64%,74%] for LDL. For all three outcomes, precision decreased only gradually as more individuals were classified as risk.

Considering the different prevalence rates, the performance was qualitatively similar compared to the testing data (the share of the 20% of the CARRS data not used for training), as shown in Supplementary Table 6. The models performed relatively better on lower parts of the risk score distribution for failure to achieve HbA1c control, and relatively poorer on top percentiles for failure to achieve SBP control, LDL control, SBP improvement, and LDL improvement. Compared to the time-shifted data, the performance was relatively poorer for the top percentiles for failure to achieve SBP or LDL control, but better for the top percentiles for failure to achieve HbA1c or SBP improvements (Supplementary Table 7 and Supplementary Fig. 5).

### Targeting based on an absolute cutoff of 50%

Alternatively, the model risk score can be used to target patients based on a fixed cutoff, e.g., targeting all patients with an absolute risk score above 50%. Table 3 displays the performance of the selected models for the validation data when using a cutoff of 50% (respectively the decision boundary for SVM algorithms). For most outcomes, the performance measures were comparable to targeting the top 50% of the sample.

**Table 3:** Performance of the machine learning predictions when patients are targeted according to the calibration cutoff

Outcome definition	Not achieving control			Not achieving improvements		
CVD risk factor	HbA1c	SBP	LDL	HbA1c	SBP	LDL
Chosen specification	Logistic (M)	Logistic (S)	Logistic (M)	Logistic (L)	Tree (S)	Logistic (M)
Detection prevalence	36%	16%	27%	69%	74%	67%
[95% CI]	[31%,41%]	[12%,20%]	[23%,32%]	[64%, 73%]	[70%, 78%]	[63%,72%]
Precision	75%	30%	23%	71%	71%	74%
[95% CI]	[67%,82%]	[19%,41%]	[15%,30%]	[66%, 77%]	[66%, 76%]	[68%,79%]
Sensitivity	53%	26%	42%	82%	88%	86%
[95% CI]	[46%,60%]	[16%,37%]	[29%,55%]	[76%, 86%]	[83%, 92%]	[82%,90%]

This is the performance of the models with the highest median area under the curve over all cross-validation folds applied to the validation data. Detection prevalence is the percentage of patients which will be selected based on the model. Precision is the percentage of selected patients not achieving the outcome. Sensitivity is the percentage of patients not achieving the outcome who are selected. Confidence intervals obtained from bootstrapping with 1000 draws.

For failure to achieve HbA1c control, the model classified 36% [31%,41%] of individuals as at risk (detection prevalence), and among these 36%, 75% [67%,82%] were correctly identified as at risk (precision). Overall, the model correctly classified 53% [46%,60%] of those failing to achieve control as at risk (sensitivity). For failure to achieve SBP control, the model classified 16% [12%,20%] of individuals as at risk, and among these, 30% [19%,41%] were correctly identified as at risk. Overall, 26% [16%,37%] of those failing to achieve were correctly classified. For failure to achieve LDL control, the model classified 27% [23%,32%] of individuals as at risk, and among these, 23% [15%,30%] were correctly identified as at risk. The model correctly classified 42% [29%,55%] of those failing to achieve control.

For failure to achieve meaningful reductions, detection prevalence, precision, and sensitivity were similar to the failure to achieve control for HbA1c, but higher for SBP and LDL. For failure to achieve meaningful reductions in HbA1c levels, the model classified 69% [64%,73%] of the sample as at risk, among which 71% [66%,77%] actually failed to achieve meaningful reductions in HbA1c. It correctly classified 82% [76%,86%] of those failing to achieve reductions. For failure to achieve meaningful reductions in SBP levels, the model classified 74% [70%,78%] of the sample as at risk, among which 71% [66%,76%] actually failed to achieve meaningful reductions in SBP. It correctly classified 88% [83%,92%] of those failing to achieve reductions. For failure to achieve meaningful reductions in LDL levels, the model classified 67% [63%,72%] of the sample as at risk, among which 74% [68%,79%] actually failed to achieve meaningful reductions in LDL. It correctly classified 86% [82%,90%] of those failing to achieve reductions.

Considering the different performance rates, the precision was similar to the testing data (Supplementary Table 6), and lower for SBP and LDL control than in the time-shifted data (Supplementary Table 7). Relatively fewer patients were classified as at risk for failure to achieve HbA1c control, SBP control, LDL control, and SBP improvement, compared to the testing data, but the share was relatively similar to the time-shifted data. Overall, sensitivity in the validation data was lower for failure to achieve control, and higher for failure to achieve improvements, compared to the testing data, and lower for SBP and LDL control but higher for HbA1c control compared to the time-shifted data.

### Differences by sociodemographic groups

**Fig. 4:**
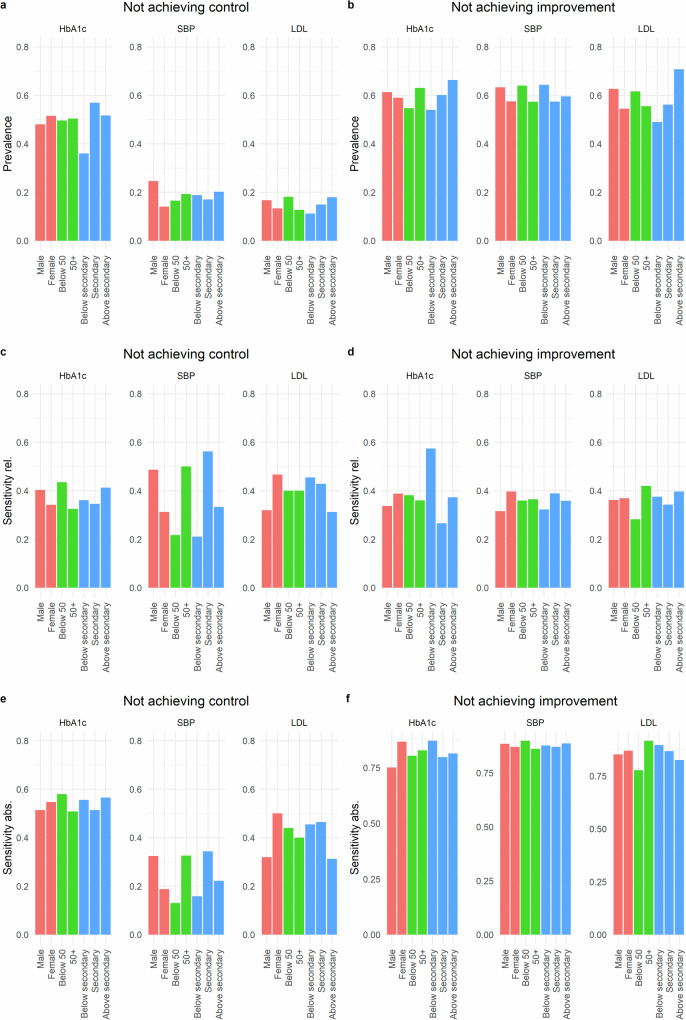
Prevalence by sociodemographic group and sensitivity of the machine learning predictions when using a relative or an absolute cutoff for targeting. Red bars indicate grouping by sex (male/female), green bars indicate grouping by age (below age 50/age 50 and older), blue bars indicate grouping by education (above secondary education, secondary education, below secondary education). **a** Prevalence for not achieving control. **b** Prevalence for not achieving improvement. **c** Sensitivity for not achieving control, targeting top 25%. **d** Sensitivity for not achieving improvement, targeting top 25%. **e** Sensitivity for not achieving control, absolute targeting. **f** Sensitivity for not achieving control, absolute targeting.

Figure 4 depicts the prevalence and sensitivity across sex, age, and education level when using a relative or an absolute risk score cutoff. There was considerable variation in the sensitivity across groups, particularly when using the relative cutoff and for not achieving CVD care goals. The most prominent differences occurred for failure to achieve SBP control: the sensitivity for female patients was about 60% of the sensitivity for male patients, the sensitivity for patients below age 50 was less than half of the sensitivity for older patients, and the sensitivity for patients with no or primary education was less than half of the sensitivity for patients with secondary education when using relative or absolute targeting. Thus, those individuals would be under selected relative to other patients if classified based on the model. For other sociodemographic outcomes and groups, differences were generally smaller.

## Discussion

Using machine learning approaches, we are able to reliably predict the risk of not meeting CVD risk factor care goals based on minimal and commonly collected clinical data for individuals receiving care. The resulting models can be used by clinicians and health administrators to efficiently identify which individuals would benefit from additional interventions to encourage risk factor control in circumstances with constrained resources where not all individuals can feasibly receive additional support. Importantly, since our models rank individuals in terms of their risk of not meeting goals, clinicians can flexibly match the share of patients that receive support with their available resources.

Our analyses show that the ranking of risk scores (relative targeting) yields more stable results compared to absolute targeting where individuals are classified based on their absolute risk. When comparing the performance across sociodemographic groups, performance differences were more pronounced for relative targeting, but these differences varied across outcomes and groups. While all models were restricted to data already collected or easily retrievable in a CVD care setting, we see that the models using only regularly collected, basic clinical data performed better or nearly as good as more comprehensive models. This suggests that these models can be used in a wide variety of clinical settings without the need to collect large amounts of data.

Similarly, the variable importance measures show that very basic patient information drive the predicted risk scores. For HbA1c outcomes, baseline CVD risk factors, age, and prevalence of neuropathy were among the most important predictors, for SBP, baseline CVD risk factors and age were important predictors, and for LDL, baseline CVD risk factors and prevalence of neuropathy were important predictors. This may indicate that current health status, together with age, play a role for achieving care goals. However, the models are not designed to detect causal patterns and this interpretation should be viewed cautiously as hypothesis generating and not as clear causal evidence.

Risk scores have become a common approach to select patients into testing or treatment recommendations. A popular example is the ACC/AHA ASCVD Risk Score, which predicts the incidence of CVD events in the upcoming 10 years^10^. Subsequent studies recalibrated the risk score or developed similar scores for other populations^14–19^. Our models are similar to these established risk score calculators in several aspects: the models can be transformed into a form where practitioners can submit the patient data and obtain risk scores or a prompt to enroll the patient in a more intensive care intervention. In the presence of electronic health record (EHR) systems, the models could be integrated into the EHR systems, such that clinicians would not need to enter data in an additional tool, but would be provided with a prompt directly from the system. In this scenario, the models could also be calibrated to the specific clinic setting and improved further based on local EHR data. Moreover, as the small models use only basic biomarkers and sociodemographic information, and perform similarly to more complex models, our models might be integrated in existing EHR systems without collecting additional patient data. This is especially relevant as availability of resources and health information systems pose a major barrier to the adoption of existing risk scores^20^. Finally, similar to the established risk scores, our models predict who will require additional care (‘high-risk’), not which type of additional care would be most efficient for the patient (‘high-benefit’). Recent research suggests that assigning interventions based on risk alone leads to suboptimal outcomes^21^. Thus, while our models support an efficient identification of patients in need, the choice of an optimal care strategy for these patients requires evidence on individualized effects of care interventions.

We add to the existing risk scores in three ways. First, our study focuses on a care-centered outcome by predicting the failure to achieve CVD care goals among patients in care, compared to the incidence of CVD events in the general population. Although control of CVD risk factors is crucial to prevent CVD events, worldwide only 67% of individuals with diagnosed diabetes achieved glycemic control, 54% blood pressure control, and 24% received statin therapy^22^. Our findings can help to narrow these gaps by targeting the available resources efficiently towards patients at high risk. Second, our prediction time span is much shorter, 12 months instead of 10 years in common risk scores. This yields a more granular prediction with immediate relevance. Third, we shed light on patients with diabetes in South Asia, a population which has been underrepresented in previous studies. At the same time, evidence for this population is urgently needed: India and Pakistan alone comprise one fifth of the global population of people with diabetes^23^, and the ongoing demographic transition is expected to further increase the pressure on the already strained health care systems^9^. One reason for this underrepresentation is the scarcity of high quality, longitudinal clinical data, a gap we fill by using data from two trials which followed structured, similar protocols.

While the availability of high quality, longitudinal data is a rare opportunity, the data also comes with some restrictions. The models are trained with patients in urban clinics, who were selected based on their poor care outcomes, and were willing to participate in a randomized controlled trial. Hence, the models might perform differently when applied on a different population. Relatedly, as the achievement of care targets is highly dependent on the care environment, changes in what constitutes usual care, both across sites and time, is expected to affect the prediction. Also, with about 1500 patients, the dataset is comparatively small, and further efficiency gains are to be expected when training the models on a larger data basis. This is also of relevance for the performance across sociodemographic groups. Given a further expansion of EHR systems, and a sufficient harmonization of systems, larger and more heterogeneous datasets will be available to recalibrate and refine the models in the future.

To conclude, our models can provide practitioners with prompts to identify patients at risk of not meeting CVD care goals and target additional efforts towards them. Such prompts could be integrated into existing EHR systems and adjusted to the available resources. Thus, our models can support challenged health care systems in South Asia to reach major CVD care goals.

## Methods

### Sample

We used data from the CARRS (ClinicalTrials.gov: NCT01212328) and INDEPENDENT (ClinicalTrials.gov: NCT02022111) trials^11,12^. Both studies were randomized clinical trials that assessed whether quality improvement interventions could improve achievement of glucose, blood pressure, and lipid care targets among patients with poorly-controlled diabetes. The interventions involved training and supplying nonphysician care coordinators to support self-management and decision support prompts linked to electronic health records for physicians. In the INDEPENDENT trial, the intervention also included specialist case reviews. In both trials, the control group received usual care.

Both trials included patients 35 years and older who had diagnosed type 2 diabetes. The CARRS trial focused on patients with poor cardiometabolic profiles, including patients with poor glycemic control (HbA1c ≥ 8%) and either uncontrolled SBP (SBP ≥ 140 mmHg) or cholesterol (LDL ≥ 130 mg/dl) or both^24^. The primary outcome was the proportion of patients achieving HbA1c < 7% and at least one of SBP < 130/80 mmHg or LDL < 100 mg/dl. The final sample included 1,146 patients from ten urban clinics in India and Pakistan, enrolled from January 2011 to June 2012. The intervention was active for 30 months. All patients were followed up at 12, 24, and 30 months after randomization, with the last visit completed in July 2014.

The INDEPENDENT trial focused on patients with diabetes and moderate to severe depression symptoms (9-item Patient Health Questionnaire score≥10) and at least one of: poor glycemic control (HbA1c ≥ 8%), SBP control (SBP ≥ 140 mmHg) or cholesterol control (LDL ≥ 130 mg/dl)^13^. The trial comprised of 404 patients from four clinics in India, enrolled from March 2015 to May 2016. The intervention was active for 12 months (the intervention group received usual care thereafter). The patients were followed-up every six months until the endline after 24 months, with the last visit completed in July 2018.

Both trials were already evaluated with respect to the impact of the interventions^11,12^. We use data from both trials as they provide a rare and novel source of longitudinal patient data in South Asia, including measured biomarkers. Both trials followed similar data collection protocols, such that we could harmonize a large set of the characteristics across both sources. Combined, this led to a database of 1550 patients. We excluded 48 patients due to missing data on main predictors, yielding a final sample of 1502 patients, with varying analyses sample sizes due to missing information on outcomes as described below. The CARRS data served as training and testing set, as described below, and the INDEPENDENT data as validation set.

### Outcomes

We focused our analysis on three main outcomes, each of them independently: Failure to achieve glycemic control (HbA1c ≥ 8%), failure to achieve hypertensive control (SBP ≥ 140 mmHg), and failure to achieve lipid control (LDL ≥ 130 mg/dl). As secondary outcomes, we considered failure to achieve clinically meaningful improvements rather than set goals (failure to reduce HbA1c by ≥1 percentage point, failure to reduce SBP by ≥10 mmHg, and failure to reduce LDL by ≥10 mg/dl), each of them independently. Both sets of outcome measures were assessed approximately 12 months after the baseline. We additionally assess the outcomes at 24 months in a sensitivity analysis. All outcomes were collected by medical staff within the original trials, and blood samples analyzed by local laboratories with external quality assurance. Due to missing information on the outcomes, the analyses samples slightly differ in their size, with 1399 patients for the HbA1c outcomes (missings: 103), 1401 patients for the SBP outcomes (missings: 101), and 1394 patients for the LDL outcomes (missings: 108).

### Predictors

The predictors are based on the data collected during the baseline of both trials, with blood samples analyzed by laboratories with external quality assurance. From this data, we selected the predictors in several steps: First, we excluded predictors only available in one of the two datasets. Next, we excluded predictors that cannot be collected easily or inexpensively in a routine care setting (e.g., extensive laboratory tests), or that might be more prone to self-report measurement errors (e.g., family history of diabetes). Finally, we excluded predictors that were only available for few respondents (e.g., costs for clinical visits). We did not include a treatment indicator as predictor, as both trials randomized treatment, and hence it should be uncorrelated with the selected predictors and should not affect the prediction validity of our models (however, the treatment might impact the external validity of our models, as discussed above).

We grouped the predictors into three different sets, as depicted in Fig. 1. The first set consists of patient information (age, gender, years since diabetes diagnosis) and basic biomarkers. The first set represents predictors that could be easily and quickly assessed during a health care visit. The second set additionally includes information about the patient’s disease history. While this set could improve predictions of who will fail to achieve CVD risk factor goals, it would also take more time to assess these factors in real world settings, and they might be more likely to suffer from measurement error. The third set additionally includes socioeconomic factors which might affect the patient’s capacity to implement the necessary behavioral steps to achieve CVD control. Finally, we screened predictors for high correlations. If two or more predictors were highly correlated (rho >0.8), we excluded predictors with less clinical relevance (based on assessments from the clinician authors of the paper) and/or the predictor that would be more challenging to collect as part of routine clinical practice in resource-poor settings. This led to the omission of total cholesterol as a predictor.

### Statistical analyses

We defined four different datasets: First, the training data, a random subsample of 80% of the CARRS data, with the random split done separately for each outcome and defined such that classes (positive/negative) were balanced across subsamples. Second, the testing data, the remaining 20% sample of the CARRS data. Third, the validation data, comprised of the INDEPENDENT sample. Fourth, the time-shifted data, using the predictors after 12 months and the outcomes after 24 months (i.e., a shift by 12 months).

We first applied three different machine learning algorithms for each of the six outcomes on the training data: gradient boosting with trees, gradient boosting with logistic regression models, and support vector machines, as implemented in the caret package in R^25^. We ran each algorithm with three sets of predictors (the small, medium, and large predictor sets described in Fig. 1). This resulted in 54 total specifications.

For each specification, we ran a 10-fold cross-validation for hyperparameter tuning for each algorithm (boosted trees: number of trees and interaction depth; boosted logistic models: number of iterations; support vector machine: cost) such that the AUC was maximized. The predictors were standardized and the observations weighted such that the classes (failure to achieve control vs. achieving control, failure to achieve reductions vs. achieving reductions) were balanced. Within each outcome, cross-validation folds were kept identical across algorithms and models. For space constraints, we present only the preferred specification for each outcome in the main body of the paper, which is the algorithm-predictor set combination with the largest median AUC across all CV-folds of the training set (see Supplementary Note 1, Supplementary Table 2, and Supplementary Fig. 1). The performance of all specifications can be found in the Supplementary Tables 3–5.

After identifying the best-performing specification for each outcome, we applied the models on the testing, validation, and time-shifted data to obtain predicted risk scores (ranging from 0% to 100%) that a patient will not achieve risk factor control/meaningful reductions for each of the CVD risk factors. We assessed the performance of these predicted risk scores in three different ways. First, we determined the observed risk of failing to meet CVD risk factor goals within 10-percentage-points bins of the predicted risk score (e.g., the observed risk among those with a predicted risk between 0–10%, 10–20%, 20–30%, etc.) and calculated the correlation between the observed and predicted risks. Second, we calculated the Brier score as the mean squared difference between the predicted risk score and the observed outcome. Third, we used the predicted risk scores to classify individuals as either at risk or not (binary classification) using cutoffs based on both their relative predicted risk score (top 50%, top 25%, and top 10%) and the commonly used threshold for classification based on the absolute predicted risk score (boosted logistic models and boosted trees: predicted risk greater than 50%, support vector machine: decision boundary). Based on this classification, we estimated the precision (what proportion of individuals classified as at risk actually failed to achieve CVD risk factor goals), sensitivity (the proportion of those that actually failed to achieve risk factor goals that were correctly classified by the model), and, in the case of absolute targeting, detection prevalence (the proportion of individuals classified as at risk). In addition, we assessed the performance of the models across sociodemographic groups. Confidence intervals were calculated using bootstrapping with 1000 draws.

Data preparation was done in Stata 17, all analyses were done in R.

### Reporting guidelines

We followed the Transparent Reporting of a multivariable prediction model for Individual Prognosis Or Diagnosis (TRIPOD) statement^26^, except for the following deviation due to the application of machine learning algorithms: Instead of presenting the full prediction model, including coefficients (item 15a), we present the variable importance measures in Fig. 2, as the algorithms do not produce traditional coefficient estimates. The TRIPOD checklist can be found in Supplementary Table 1.

### Inclusion and ethics

The studies on which the data is based included local researchers throughout the research process, which are also coauthors of the current manuscript. Access to this data has been granted by the initial research teams, including local researchers. The research is highly relevant to the local context and was determined in collaboration with the local partners. The roles and responsibilities were agreed upon ahead of the research. No capacity building was planned. This research would not have been severely restricted in the setting of the non-local researchers. The original data collections were approved by local ethics review committees (Madras Diabetes Research Foundation, All India Institute of Medical Sciences, Endocrine and Diabetes Center, Diacon Hospital, Bangalore Endocrinology and Diabetes Research Centre, St. John’s Medical College & Hospital, Diabetes Research Centre & MV Hospital for Diabetes, Public Health Foundation of India, Goa Medical College, CARE Hospital, Osmania General Hospital, Amrita Institute of Medical Sciences, Topiwala National Medical College & BYL Nair Ch. Hospital, The Aga Khan University) and the coordinating centers (Madras Diabetes Research Foundation, Public Health Foundation of India, and Emory University). The personal risk to the participants or researchers beyond the common risks of regular diabetes care were minimal. Participants gave written informed consent. Local and regional research was cited where relevant.

## Supplementary information

Supplementary Information

## Data Availability

The data that support the findings of this study are available from the INDEPENDENT and CARRS trial groups but restrictions apply to the availability of these data, which were used under license for the current study, and so are not publicly available. Data are however available from the authors upon reasonable request and with permission of the INDEPENDENT and CARRS trial groups.

## Code availability

The underlying code for this study is available at https://osf.io/289jn/.
