# Supplementary material for: Predicting control of cardiovascular disease risk factors in South Asia using machine learning
Source: NPJ Digit Med. 2024 Dec 10;7:357. doi: 10.1038/s41746-024-01353-9 (PMC11631980; doi:10.1038/s41746-024-01353-9)
Supplement: Supplementary file 1 — Supplementary Information [file 41746_2024_1353_MOESM1_ESM.pdf]

Supplementary Information

Supplementary Note 1. Model calibration ... 3

Supplementary Note 2. Performance on the testing set ... 21

Supplementary Note 3. Sensitivity analysis based on different cutoffs ... 22

Supplementary Note 4. Sensitivity analyses based on different timing ... 24

Supplementary Figure 1 ... 4

Supplementary Figure 2 ... 21

Supplementary Figure 3 ... 23

Supplementary Figure 4 ... 25

Supplementary Figure 5 ... 26

Supplementary Table 1. TRIPOD checklist ... 2

Supplementary Table 2. Model parameters of the selected models ... 3

Supplementary Table 3. Parameters and performance across CV folds for all specifications using boosted logistic regression ... 5

Supplementary Table 4. Parameters and performance across CV folds for all specifications using boosted trees ... 9

Supplementary Table 5. Parameters and performance across CV folds for all specifications using support vector machines ... 19

Supplementary Table 6. Performance for the testing set ... 22

Supplementary Table 7. Performance for different timing ... 24

Supplementary Table 1. TRIPOD checklist

Section/Topic	Item	Checklist Item	Page
Title and abstract			
Title	1	Identify the study as developing and/or validating a multivariable prediction model, the target population, and the outcome to be predicted.	1
Abstract	2	Provide a summary of objectives, study design, setting, participants, sample size, predictors, outcome, statistical analysis, results, and conclusions.	1
Introduction			
Background and objectives	3a	Explain the medical context (including whether diagnostic or prognostic) and rationale for developing or validating the multivariable prediction model, including references to existing models.	2
	3b	Specify the objectives, including whether the study describes the development or validation of the model or both.	2
Methods			
Source of data	4a	Describe the study design or source of data (e.g., randomized trial, cohort, or registry data), separately for the development and validation data sets, if applicable.	10
	4b	Specify the key study dates, including start of accrual; end of accrual; and, if applicable, end of follow-up.	11
Participants	5a	Specify key elements of the study setting (e.g., primary care, secondary care, general population) including number and location of centres.	10-11
	5b	Describe eligibility criteria for participants.	10-11
	5c	Give details of treatments received, if relevant.	10-11
Outcome	6a	Clearly define the outcome that is predicted by the prediction model, including how and when assessed.	11
	6b	Report any actions to blind assessment of the outcome to be predicted.	11
Predictors	7a	Clearly define all predictors used in developing or validating the multivariable prediction model, including how and when they were measured.	12
	7b	Report any actions to blind assessment of predictors for the outcome and other predictors.	12
Sample size	8	Explain how the study size was arrived at.	11-12
Missing data	9	Describe how missing data were handled (e.g., complete-case analysis, single imputation, multiple imputation) with details of any imputation method.	11-12
Statistical analysis methods	10a	Describe how predictors were handled in the analyses.	13
	10b	Specify type of model, all model-building procedures (including any predictor selection), and method for internal validation.	12-13
	10d	Specify all measures used to assess model performance and, if relevant, to compare multiple models.	13
Risk groups	11	Provide details on how risk groups were created, if done.	13
Results			
Participants	13a	Describe the flow of participants through the study, including the number of participants with and without the outcome and, if applicable, a summary of the follow-up time. A diagram may be helpful.	11-12
	13b	Describe the characteristics of the participants (basic demographics, clinical features, available predictors), including the number of participants with missing data for predictors and outcome.	Table 1
Model development	14a	Specify the number of participants and outcome events in each analysis.	Table 2
	14b	If done, report the unadjusted association between each candidate predictor and outcome.	NA
Model specification	15a	Present the full prediction model to allow predictions for individuals (i.e., all regression coefficients, and model intercept or baseline survival at a given time point).	NA[1]
	15b	Explain how to the use the prediction model.	8-10
Model performance	16	Report performance measures (with CIs) for the prediction model.	Tables 2 & 3
Discussion			
Limitations	18	Discuss any limitations of the study (such as nonrepresentative sample, few events per predictor, missing data).	9-10
Interpretation	19b	Give an overall interpretation of the results, considering objectives, limitations, and results from similar studies, and other relevant evidence.	8-10
Implications	20	Discuss the potential clinical use of the model and implications for future research.	9-10
Other information			
Supplementary information	21	Provide information about the availability of supplementary resources, such as study protocol, Web calculator, and data sets.	15
Funding	22	Give the source of funding and the role of the funders for the present study.	15

Notes: [1]As the algorithms do not produce traditional coefficient estimates, variable importance measures are presented in Figure 2.

Supplementary Note 1. Model calibration

For each outcome, we create nine models: One for each combination of machine learning algorithms, boosted logistic model ("logistic"), boosted tree ("tree"), support vector machines ("SVM") with the set of predictors, small ("S"), medium-sized ("M"). and large ("L"), with the predictor sets described in Figure 1 in the main paper. The models were calibrated across the 10-fold cross-validation and their performance assessed (an overview is shown in Supplementary Figure 1). In general, there are no large differences in performances within each outcome. The model parameters of the best performing models are shown in Supplementary Table 2. A full list of all models and their performance across the 10 CV folds can be found in Supplementary Table 3, Supplementary Table 4, and Supplementary Table 5.

For the risk of not achieving HbA1c control, the medium-sized boosted logistic model performed best, followed by the medium-sized boosted tree model. For SBP and small boosted logistic model performed best, followed by the medium boosted logistic model. For LDL, the medium boosted logistic model performed best, followed by the large support vector machine model. When training the models to predict a meaningful improvement, the large boosted logistic model performs best for HbA1c, followed by the medium boosted logistic model. For SBP, the small boosted tree model performs best, followed by the large boosted logistic model. For LDL, the medium-sized boosted logistic model performs best, followed by the large model.

Supplementary Table 2. Model parameters of the selected models

Outcome	Parameters
HbA1c control	Boosting iterations: 100
SBP control	Boosting iterations: 200
LDL control	Boosting iterations: 50
HbA1c improvement	Boosting iterations: 150
SBP improvement	Trees: 50, interaction depth: 4
LDL improvement	Boosting iterations: 100

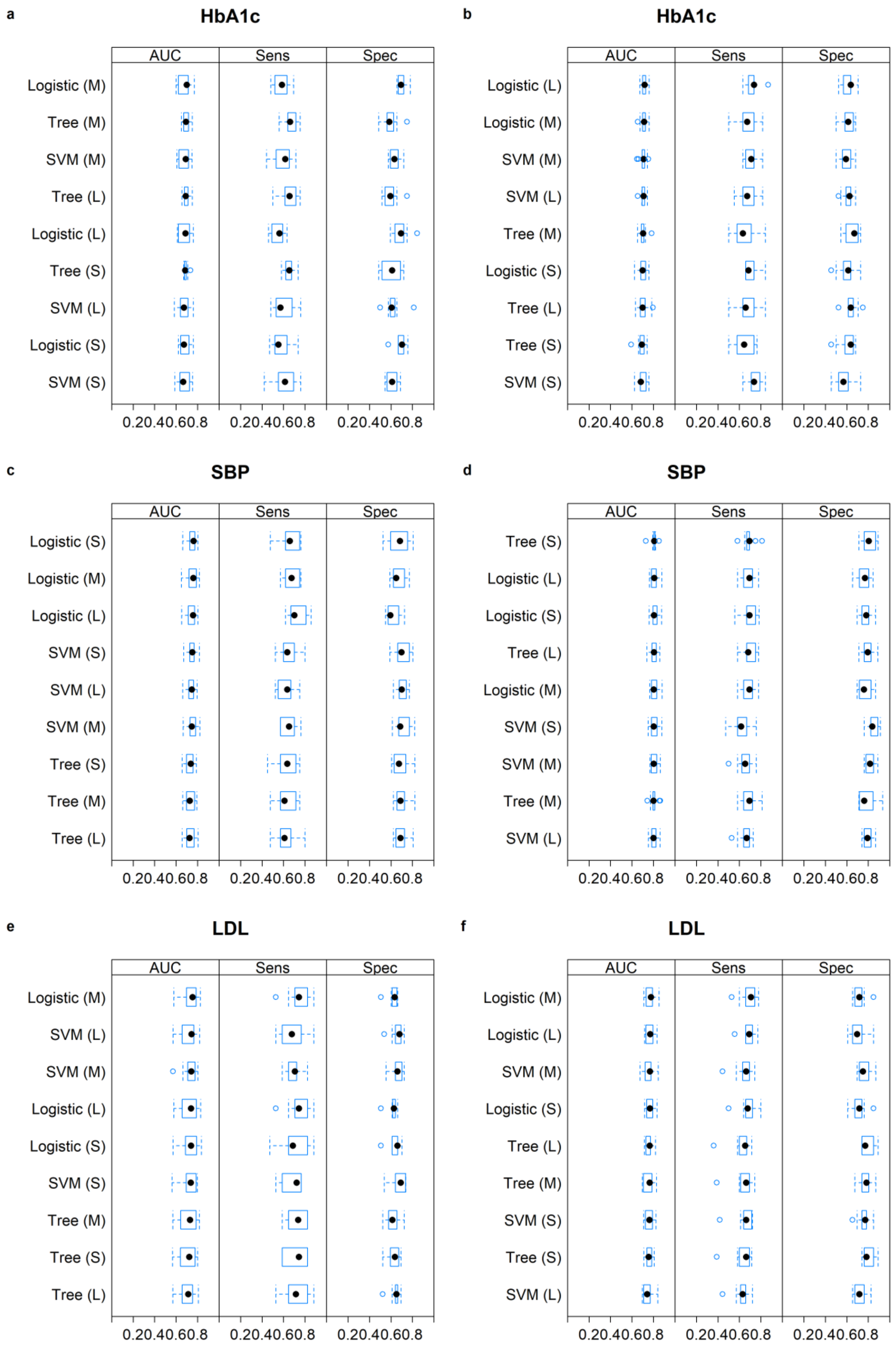

Supplementary Figure 1. Performance across CV-folds. Boxplots for the area under the curve (AUC), sensitivity (Sens), and specificity (Spec) across all CV-folds. a Not achieving HbA1c control. b Not achieving HbA1c improvement. c Not achieving SBP control. d Not achieving SBP improvement. e Not achieving LDL control. f Not achieving LDL improvement.

Supplementary Table 3. Parameters and performance across CV folds for all specifications using boosted logistic regression

Outcome	Definition	Predictors	Boosting iterations	AUC (SD)	Sensitivity (SD)	Specificity (SD)
HbA1c	control	S	50	68% (4%)	57% (8%)	69% (5%)
HbA1c	control	S	100	68% (5%)	59% (8%)	67% (7%)
HbA1c	control	S	150	68% (5%)	59% (9%)	65% (7%)
HbA1c	control	S	200	68% (5%)	59% (8%)	64% (7%)
HbA1c	control	S	250	67% (5%)	60% (8%)	63% (7%)
HbA1c	control	S	300	67% (5%)	60% (8%)	63% (7%)
HbA1c	control	S	350	67% (5%)	60% (8%)	62% (6%)
HbA1c	control	S	400	67% (5%)	60% (8%)	62% (6%)
HbA1c	control	S	450	67% (5%)	60% (9%)	62% (6%)
HbA1c	control	S	500	67% (5%)	60% (9%)	62% (6%)
HbA1c	control	M	50	68% (5%)	56% (6%)	70% (6%)
HbA1c	control	M	100	68% (5%)	58% (7%)	70% (4%)
HbA1c	control	M	150	68% (6%)	60% (8%)	68% (3%)
HbA1c	control	M	200	68% (6%)	60% (9%)	67% (4%)
HbA1c	control	M	250	68% (6%)	60% (8%)	67% (4%)
HbA1c	control	M	300	68% (6%)	61% (9%)	66% (5%)
HbA1c	control	M	350	68% (6%)	61% (9%)	66% (5%)
HbA1c	control	M	400	68% (6%)	61% (8%)	66% (5%)
HbA1c	control	M	450	68% (6%)	61% (8%)	65% (5%)
HbA1c	control	M	500	68% (6%)	61% (8%)	66% (6%)
HbA1c	control	L	50	68% (5%)	55% (6%)	69% (7%)
HbA1c	control	L	100	68% (6%)	57% (7%)	69% (5%)
HbA1c	control	L	150	67% (6%)	58% (8%)	66% (6%)
HbA1c	control	L	200	67% (6%)	59% (8%)	66% (7%)
HbA1c	control	L	250	67% (6%)	60% (8%)	65% (7%)
HbA1c	control	L	300	67% (6%)	60% (8%)	65% (7%)
HbA1c	control	L	350	67% (6%)	60% (8%)	64% (7%)
HbA1c	control	L	400	67% (6%)	60% (8%)	63% (7%)
HbA1c	control	L	450	67% (6%)	61% (8%)	63% (7%)
HbA1c	control	L	500	67% (6%)	61% (8%)	63% (7%)
SBP	control	S	50	74% (5%)	72% (10%)	62% (8%)
SBP	control	S	100	75% (5%)	68% (10%)	66% (9%)
SBP	control	S	150	75% (5%)	67% (10%)	67% (9%)
SBP	control	S	200	75% (4%)	66% (9%)	67% (9%)
SBP	control	S	250	75% (4%)	66% (9%)	68% (9%)
SBP	control	S	300	75% (4%)	66% (9%)	68% (9%)
SBP	control	S	350	75% (4%)	66% (9%)	69% (8%)
SBP	control	S	400	75% (4%)	66% (8%)	68% (8%)
SBP	control	S	450	75% (4%)	66% (8%)	68% (8%)
SBP	control	S	500	75% (4%)	66% (8%)	68% (8%)
SBP	control	M	50	75% (4%)	74% (10%)	63% (6%)
SBP	control	M	100	75% (5%)	70% (10%)	65% (8%)
SBP	control	M	150	75% (5%)	68% (8%)	66% (7%)
SBP	control	M	200	75% (5%)	68% (8%)	67% (6%)
SBP	control	M	250	75% (5%)	68% (8%)	68% (7%)

SBP	control	M	300	75% (5%)	67% (7%)	68% (7%)
SBP	control	M	350	75% (5%)	67% (7%)	68% (6%)
SBP	control	M	400	75% (5%)	67% (7%)	69% (7%)
SBP	control	M	450	75% (5%)	67% (7%)	69% (7%)
SBP	control	M	500	75% (5%)	67% (7%)	69% (7%)
SBP	control	L	50	74% (5%)	73% (9%)	62% (7%)
SBP	control	L	100	74% (4%)	68% (8%)	65% (8%)
SBP	control	L	150	74% (4%)	67% (9%)	66% (7%)
SBP	control	L	200	74% (4%)	66% (8%)	66% (7%)
SBP	control	L	250	74% (4%)	65% (7%)	67% (8%)
SBP	control	L	300	74% (4%)	65% (7%)	67% (8%)
SBP	control	L	350	74% (4%)	65% (7%)	67% (8%)
SBP	control	L	400	74% (4%)	65% (7%)	67% (8%)
SBP	control	L	450	74% (4%)	66% (8%)	68% (8%)
SBP	control	L	500	74% (4%)	66% (8%)	67% (7%)
LDL	control	S	50	73% (8%)	70% (12%)	64% (6%)
LDL	control	S	100	72% (8%)	69% (10%)	66% (6%)
LDL	control	S	150	72% (8%)	68% (9%)	67% (5%)
LDL	control	S	200	72% (8%)	67% (9%)	68% (6%)
LDL	control	S	250	72% (8%)	67% (9%)	68% (6%)
LDL	control	S	300	72% (8%)	67% (9%)	68% (6%)
LDL	control	S	350	72% (8%)	67% (9%)	68% (6%)
LDL	control	S	400	72% (8%)	68% (9%)	68% (6%)
LDL	control	S	450	72% (8%)	68% (9%)	68% (6%)
LDL	control	S	500	72% (8%)	67% (9%)	68% (6%)
LDL	control	M	50	74% (8%)	74% (10%)	62% (5%)
LDL	control	M	100	73% (7%)	73% (11%)	66% (5%)
LDL	control	M	150	73% (7%)	72% (11%)	67% (5%)
LDL	control	M	200	73% (7%)	70% (11%)	68% (5%)
LDL	control	M	250	73% (7%)	69% (10%)	69% (5%)
LDL	control	M	300	73% (7%)	69% (9%)	69% (5%)
LDL	control	M	350	73% (7%)	69% (11%)	69% (6%)
LDL	control	M	400	73% (7%)	69% (10%)	69% (6%)
LDL	control	M	450	73% (7%)	69% (10%)	69% (6%)
LDL	control	M	500	73% (7%)	69% (9%)	69% (6%)
LDL	control	L	50	73% (8%)	74% (10%)	62% (4%)
LDL	control	L	100	72% (8%)	70% (11%)	65% (5%)
LDL	control	L	150	72% (7%)	69% (12%)	67% (5%)
LDL	control	L	200	72% (7%)	67% (11%)	68% (5%)
LDL	control	L	250	72% (7%)	67% (11%)	68% (5%)
LDL	control	L	300	72% (7%)	68% (11%)	68% (5%)
LDL	control	L	350	72% (7%)	67% (11%)	68% (5%)
LDL	control	L	400	72% (7%)	66% (11%)	68% (5%)
LDL	control	L	450	72% (7%)	66% (11%)	68% (5%)
LDL	control	L	500	72% (7%)	66% (11%)	68% (5%)
HbA1c	improvement	S	50	70% (5%)	73% (6%)	57% (8%)
HbA1c	improvement	S	100	70% (5%)	73% (6%)	59% (8%)

HbA1c	improvement	S	150	70% (4%)	71% (6%)	60% (8%)
HbA1c	improvement	S	200	70% (4%)	71% (6%)	61% (8%)
HbA1c	improvement	S	250	70% (4%)	69% (7%)	61% (8%)
HbA1c	improvement	S	300	70% (4%)	69% (7%)	61% (8%)
HbA1c	improvement	S	350	70% (4%)	69% (8%)	61% (8%)
HbA1c	improvement	S	400	70% (4%)	69% (8%)	61% (8%)
HbA1c	improvement	S	450	70% (4%)	69% (8%)	61% (8%)
HbA1c	improvement	S	500	70% (4%)	69% (8%)	61% (8%)
HbA1c	improvement	M	50	71% (3%)	74% (6%)	58% (6%)
HbA1c	improvement	M	100	71% (3%)	74% (7%)	60% (6%)
HbA1c	improvement	M	150	71% (3%)	71% (7%)	60% (5%)
HbA1c	improvement	M	200	71% (3%)	69% (8%)	61% (6%)
HbA1c	improvement	M	250	71% (3%)	68% (9%)	61% (6%)
HbA1c	improvement	M	300	71% (3%)	68% (9%)	62% (6%)
HbA1c	improvement	M	350	71% (3%)	68% (9%)	63% (6%)
HbA1c	improvement	M	400	71% (3%)	68% (9%)	62% (6%)
HbA1c	improvement	M	450	71% (3%)	67% (9%)	62% (6%)
HbA1c	improvement	M	500	71% (3%)	67% (8%)	62% (6%)
HbA1c	improvement	L	50	71% (3%)	74% (5%)	60% (6%)
HbA1c	improvement	L	100	71% (3%)	74% (7%)	61% (7%)
HbA1c	improvement	L	150	71% (3%)	72% (6%)	61% (6%)
HbA1c	improvement	L	200	71% (3%)	71% (7%)	62% (5%)
HbA1c	improvement	L	250	71% (2%)	69% (7%)	62% (5%)
HbA1c	improvement	L	300	71% (3%)	70% (7%)	63% (6%)
HbA1c	improvement	L	350	71% (2%)	69% (7%)	63% (6%)
HbA1c	improvement	L	400	71% (2%)	69% (7%)	62% (6%)
HbA1c	improvement	L	450	71% (2%)	69% (7%)	63% (5%)
HbA1c	improvement	L	500	71% (2%)	68% (7%)	63% (5%)
SBP	improvement	S	50	81% (4%)	69% (6%)	78% (5%)
SBP	improvement	S	100	81% (4%)	69% (6%)	77% (6%)
SBP	improvement	S	150	81% (4%)	70% (6%)	77% (6%)
SBP	improvement	S	200	81% (4%)	70% (7%)	78% (6%)
SBP	improvement	S	250	81% (4%)	70% (7%)	78% (6%)
SBP	improvement	S	300	81% (4%)	70% (7%)	77% (6%)
SBP	improvement	S	350	81% (4%)	70% (7%)	77% (6%)
SBP	improvement	S	400	81% (4%)	70% (7%)	78% (5%)
SBP	improvement	S	450	81% (4%)	70% (7%)	78% (6%)
SBP	improvement	S	500	82% (4%)	70% (7%)	77% (5%)
SBP	improvement	M	50	81% (4%)	68% (7%)	78% (7%)
SBP	improvement	M	100	81% (4%)	69% (6%)	76% (8%)
SBP	improvement	M	150	81% (4%)	68% (6%)	76% (7%)
SBP	improvement	M	200	81% (4%)	68% (6%)	76% (7%)
SBP	improvement	M	250	81% (4%)	69% (6%)	76% (6%)
SBP	improvement	M	300	81% (4%)	69% (6%)	76% (6%)
SBP	improvement	M	350	81% (4%)	69% (6%)	76% (6%)
SBP	improvement	M	400	81% (4%)	70% (6%)	76% (6%)
SBP	improvement	M	450	81% (4%)	70% (6%)	76% (6%)

SBP	improvement	M	500	81% (4%)	70% (6%)	77% (5%)
SBP	improvement	L	50	80% (4%)	68% (6%)	78% (6%)
SBP	improvement	L	100	81% (4%)	69% (5%)	76% (7%)
SBP	improvement	L	150	81% (4%)	69% (6%)	75% (6%)
SBP	improvement	L	200	81% (4%)	69% (6%)	76% (6%)
SBP	improvement	L	250	81% (4%)	69% (6%)	75% (5%)
SBP	improvement	L	300	81% (3%)	68% (6%)	74% (5%)
SBP	improvement	L	350	81% (3%)	68% (6%)	75% (5%)
SBP	improvement	L	400	81% (3%)	68% (6%)	75% (5%)
SBP	improvement	L	450	81% (3%)	69% (6%)	75% (5%)
SBP	improvement	L	500	81% (3%)	69% (6%)	75% (5%)
LDL	improvement	S	50	77% (4%)	68% (8%)	71% (6%)
LDL	improvement	S	100	77% (4%)	68% (9%)	72% (6%)
LDL	improvement	S	150	77% (4%)	68% (9%)	72% (6%)
LDL	improvement	S	200	76% (4%)	67% (8%)	72% (6%)
LDL	improvement	S	250	76% (4%)	68% (7%)	72% (7%)
LDL	improvement	S	300	76% (4%)	68% (7%)	72% (7%)
LDL	improvement	S	350	76% (4%)	68% (7%)	72% (7%)
LDL	improvement	S	400	76% (4%)	68% (7%)	72% (7%)
LDL	improvement	S	450	76% (4%)	68% (7%)	72% (7%)
LDL	improvement	S	500	76% (4%)	68% (7%)	72% (7%)
LDL	improvement	M	50	77% (4%)	69% (8%)	70% (6%)
LDL	improvement	M	100	77% (4%)	69% (8%)	72% (6%)
LDL	improvement	M	150	77% (4%)	69% (7%)	72% (5%)
LDL	improvement	M	200	77% (4%)	69% (7%)	73% (5%)
LDL	improvement	M	250	77% (5%)	68% (7%)	73% (5%)
LDL	improvement	M	300	77% (5%)	68% (7%)	73% (5%)
LDL	improvement	M	350	77% (5%)	68% (7%)	73% (5%)
LDL	improvement	M	400	77% (5%)	68% (7%)	72% (5%)
LDL	improvement	M	450	76% (5%)	68% (7%)	72% (5%)
LDL	improvement	M	500	76% (5%)	68% (7%)	72% (5%)
LDL	improvement	L	50	77% (4%)	69% (6%)	70% (7%)
LDL	improvement	L	100	77% (4%)	68% (7%)	70% (5%)
LDL	improvement	L	150	76% (4%)	68% (6%)	70% (5%)
LDL	improvement	L	200	76% (4%)	68% (7%)	70% (4%)
LDL	improvement	L	250	76% (4%)	68% (7%)	70% (4%)
LDL	improvement	L	300	76% (4%)	68% (7%)	71% (4%)
LDL	improvement	L	350	76% (4%)	67% (6%)	71% (4%)
LDL	improvement	L	400	76% (4%)	68% (5%)	71% (3%)
LDL	improvement	L	450	76% (4%)	68% (5%)	71% (3%)
LDL	improvement	L	500	76% (4%)	67% (5%)	70% (4%)

Supplementary Table 4. Parameters and performance across CV folds for all specifications using boosted trees

Outcome	Definition	Predictors	Trees	Interaction depth	AUC (SD)	Sensitivity (SD)	Specificity (SD)
HbA1c	control	S	50	1	69% (2%)	65% (4%)	61% (9%)
HbA1c	control	S	50	2	69% (3%)	65% (4%)	63% (8%)
HbA1c	control	S	50	3	67% (3%)	66% (8%)	59% (10%)
HbA1c	control	S	50	4	66% (3%)	64% (9%)	59% (8%)
HbA1c	control	S	50	5	65% (3%)	64% (5%)	58% (7%)
HbA1c	control	S	100	1	68% (3%)	66% (4%)	62% (10%)
HbA1c	control	S	100	2	68% (3%)	67% (5%)	61% (8%)
HbA1c	control	S	100	3	66% (4%)	67% (6%)	58% (8%)
HbA1c	control	S	100	4	65% (3%)	65% (7%)	57% (9%)
HbA1c	control	S	100	5	63% (3%)	64% (5%)	54% (7%)
HbA1c	control	S	150	1	68% (3%)	65% (5%)	60% (9%)
HbA1c	control	S	150	2	66% (3%)	65% (5%)	60% (8%)
HbA1c	control	S	150	3	65% (5%)	65% (6%)	57% (9%)
HbA1c	control	S	150	4	63% (4%)	65% (6%)	55% (6%)
HbA1c	control	S	150	5	62% (4%)	64% (5%)	53% (9%)
HbA1c	control	S	200	1	67% (3%)	65% (5%)	58% (9%)
HbA1c	control	S	200	2	65% (4%)	65% (4%)	57% (9%)
HbA1c	control	S	200	3	64% (4%)	64% (7%)	57% (9%)
HbA1c	control	S	200	4	62% (4%)	64% (6%)	56% (8%)
HbA1c	control	S	200	5	61% (4%)	65% (5%)	50% (9%)
HbA1c	control	S	250	1	66% (4%)	66% (5%)	59% (9%)
HbA1c	control	S	250	2	64% (4%)	64% (7%)	57% (8%)
HbA1c	control	S	250	3	63% (4%)	63% (6%)	57% (10%)
HbA1c	control	S	250	4	62% (4%)	64% (5%)	54% (8%)
HbA1c	control	S	250	5	61% (4%)	66% (5%)	49% (8%)
HbA1c	control	M	50	1	69% (3%)	67% (6%)	59% (7%)
HbA1c	control	M	50	2	69% (4%)	67% (7%)	62% (5%)
HbA1c	control	M	50	3	69% (4%)	66% (6%)	61% (5%)
HbA1c	control	M	50	4	68% (4%)	66% (7%)	58% (9%)
HbA1c	control	M	50	5	66% (4%)	66% (7%)	56% (8%)
HbA1c	control	M	100	1	69% (4%)	66% (6%)	63% (6%)
HbA1c	control	M	100	2	68% (4%)	67% (6%)	59% (7%)
HbA1c	control	M	100	3	67% (4%)	65% (6%)	60% (10%)
HbA1c	control	M	100	4	67% (3%)	67% (5%)	60% (8%)
HbA1c	control	M	100	5	64% (3%)	62% (7%)	53% (8%)
HbA1c	control	M	150	1	69% (4%)	65% (5%)	61% (6%)
HbA1c	control	M	150	2	67% (4%)	67% (6%)	58% (6%)
HbA1c	control	M	150	3	66% (5%)	64% (6%)	58% (9%)
HbA1c	control	M	150	4	66% (3%)	66% (7%)	57% (7%)
HbA1c	control	M	150	5	63% (4%)	65% (5%)	51% (7%)
HbA1c	control	M	200	1	67% (4%)	67% (6%)	58% (8%)
HbA1c	control	M	200	2	66% (4%)	67% (6%)	58% (6%)
HbA1c	control	M	200	3	65% (5%)	65% (9%)	57% (6%)
HbA1c	control	M	200	4	65% (4%)	66% (4%)	57% (6%)
HbA1c	control	M	200	5	63% (3%)	65% (6%)	51% (7%)

HbA1c	control	M	250	1	67% (4%)	67% (5%)	59% (8%)
HbA1c	control	M	250	2	65% (4%)	65% (6%)	56% (7%)
HbA1c	control	M	250	3	64% (5%)	63% (8%)	57% (9%)
HbA1c	control	M	250	4	64% (3%)	66% (6%)	57% (6%)
HbA1c	control	M	250	5	63% (3%)	66% (6%)	52% (8%)
HbA1c	control	L	50	1	69% (3%)	65% (8%)	60% (7%)
HbA1c	control	L	50	2	69% (3%)	67% (7%)	62% (6%)
HbA1c	control	L	50	3	68% (4%)	67% (6%)	61% (9%)
HbA1c	control	L	50	4	68% (3%)	66% (4%)	61% (7%)
HbA1c	control	L	50	5	68% (5%)	67% (7%)	61% (9%)
HbA1c	control	L	100	1	69% (4%)	66% (6%)	62% (7%)
HbA1c	control	L	100	2	68% (4%)	66% (7%)	58% (6%)
HbA1c	control	L	100	3	67% (4%)	66% (7%)	60% (10%)
HbA1c	control	L	100	4	66% (4%)	67% (6%)	57% (8%)
HbA1c	control	L	100	5	67% (3%)	66% (6%)	56% (8%)
HbA1c	control	L	150	1	68% (4%)	65% (7%)	61% (8%)
HbA1c	control	L	150	2	67% (4%)	66% (7%)	58% (7%)
HbA1c	control	L	150	3	66% (5%)	67% (8%)	57% (9%)
HbA1c	control	L	150	4	65% (3%)	67% (6%)	55% (7%)
HbA1c	control	L	150	5	65% (3%)	66% (6%)	57% (8%)
HbA1c	control	L	200	1	68% (4%)	65% (5%)	61% (8%)
HbA1c	control	L	200	2	66% (4%)	67% (6%)	56% (8%)
HbA1c	control	L	200	3	65% (4%)	65% (7%)	58% (8%)
HbA1c	control	L	200	4	64% (3%)	67% (5%)	54% (6%)
HbA1c	control	L	200	5	65% (4%)	69% (6%)	54% (9%)
HbA1c	control	L	250	1	67% (4%)	65% (6%)	61% (8%)
HbA1c	control	L	250	2	65% (5%)	67% (8%)	56% (9%)
HbA1c	control	L	250	3	64% (4%)	64% (6%)	55% (7%)
HbA1c	control	L	250	4	63% (4%)	65% (6%)	55% (7%)
HbA1c	control	L	250	5	64% (5%)	68% (7%)	55% (9%)
SBP	control	S	50	1	73% (4%)	63% (10%)	69% (7%)
SBP	control	S	50	2	72% (5%)	63% (11%)	69% (9%)
SBP	control	S	50	3	71% (5%)	57% (11%)	70% (10%)
SBP	control	S	50	4	71% (7%)	60% (12%)	71% (7%)
SBP	control	S	50	5	70% (7%)	58% (15%)	74% (6%)
SBP	control	S	100	1	72% (5%)	63% (10%)	70% (9%)
SBP	control	S	100	2	71% (4%)	62% (7%)	70% (7%)
SBP	control	S	100	3	70% (5%)	57% (10%)	74% (7%)
SBP	control	S	100	4	69% (8%)	54% (12%)	74% (6%)
SBP	control	S	100	5	69% (7%)	51% (13%)	76% (4%)
SBP	control	S	150	1	72% (5%)	63% (12%)	69% (8%)
SBP	control	S	150	2	71% (5%)	61% (12%)	71% (7%)
SBP	control	S	150	3	69% (6%)	53% (10%)	74% (6%)
SBP	control	S	150	4	69% (8%)	49% (15%)	76% (7%)
SBP	control	S	150	5	69% (6%)	49% (14%)	77% (5%)
SBP	control	S	200	1	71% (7%)	63% (12%)	69% (8%)
SBP	control	S	200	2	70% (6%)	55% (13%)	72% (7%)

SBP	control	S	200	3	68% (7%)	47% (12%)	75% (8%)
SBP	control	S	200	4	69% (9%)	47% (11%)	78% (7%)
SBP	control	S	200	5	68% (7%)	46% (15%)	79% (5%)
SBP	control	S	250	1	71% (6%)	60% (12%)	70% (8%)
SBP	control	S	250	2	70% (6%)	54% (9%)	74% (5%)
SBP	control	S	250	3	67% (7%)	48% (10%)	75% (7%)
SBP	control	S	250	4	69% (9%)	48% (11%)	77% (7%)
SBP	control	S	250	5	69% (7%)	48% (16%)	79% (6%)
SBP	control	M	50	1	73% (4%)	62% (9%)	70% (6%)
SBP	control	M	50	2	72% (5%)	63% (10%)	71% (8%)
SBP	control	M	50	3	71% (5%)	57% (11%)	72% (8%)
SBP	control	M	50	4	71% (7%)	57% (14%)	73% (8%)
SBP	control	M	50	5	71% (6%)	58% (11%)	74% (8%)
SBP	control	M	100	1	72% (5%)	62% (11%)	70% (8%)
SBP	control	M	100	2	72% (4%)	60% (10%)	71% (7%)
SBP	control	M	100	3	71% (5%)	57% (10%)	73% (7%)
SBP	control	M	100	4	70% (7%)	52% (15%)	77% (6%)
SBP	control	M	100	5	68% (5%)	50% (13%)	75% (7%)
SBP	control	M	150	1	72% (5%)	62% (11%)	70% (7%)
SBP	control	M	150	2	71% (4%)	62% (9%)	73% (7%)
SBP	control	M	150	3	70% (5%)	54% (9%)	73% (7%)
SBP	control	M	150	4	69% (7%)	52% (12%)	76% (7%)
SBP	control	M	150	5	68% (5%)	48% (11%)	76% (6%)
SBP	control	M	200	1	72% (6%)	62% (12%)	71% (7%)
SBP	control	M	200	2	70% (6%)	60% (7%)	72% (7%)
SBP	control	M	200	3	68% (5%)	47% (10%)	74% (7%)
SBP	control	M	200	4	69% (8%)	44% (13%)	77% (8%)
SBP	control	M	200	5	67% (5%)	44% (13%)	78% (6%)
SBP	control	M	250	1	71% (5%)	61% (11%)	70% (8%)
SBP	control	M	250	2	70% (5%)	55% (9%)	73% (7%)
SBP	control	M	250	3	68% (5%)	49% (8%)	75% (5%)
SBP	control	M	250	4	68% (7%)	44% (12%)	77% (7%)
SBP	control	M	250	5	66% (6%)	44% (13%)	79% (6%)
SBP	control	L	50	1	73% (5%)	62% (9%)	70% (6%)
SBP	control	L	50	2	72% (5%)	61% (8%)	70% (8%)
SBP	control	L	50	3	71% (5%)	57% (6%)	71% (8%)
SBP	control	L	50	4	72% (5%)	59% (7%)	72% (6%)
SBP	control	L	50	5	70% (6%)	52% (14%)	73% (7%)
SBP	control	L	100	1	72% (5%)	62% (9%)	69% (7%)
SBP	control	L	100	2	71% (4%)	59% (9%)	71% (7%)
SBP	control	L	100	3	70% (4%)	54% (9%)	72% (6%)
SBP	control	L	100	4	70% (5%)	53% (13%)	74% (7%)
SBP	control	L	100	5	68% (6%)	46% (13%)	74% (5%)
SBP	control	L	150	1	72% (4%)	61% (10%)	70% (6%)
SBP	control	L	150	2	71% (4%)	58% (9%)	72% (7%)
SBP	control	L	150	3	69% (5%)	51% (11%)	74% (6%)
SBP	control	L	150	4	69% (5%)	49% (10%)	75% (6%)

SBP	control	L	150	5	67% (6%)	47% (10%)	76% (5%)
SBP	control	L	200	1	71% (6%)	61% (10%)	70% (5%)
SBP	control	L	200	2	69% (4%)	55% (8%)	71% (6%)
SBP	control	L	200	3	68% (6%)	48% (9%)	74% (4%)
SBP	control	L	200	4	69% (5%)	45% (10%)	77% (5%)
SBP	control	L	200	5	67% (6%)	45% (8%)	77% (4%)
SBP	control	L	250	1	72% (5%)	58% (10%)	70% (7%)
SBP	control	L	250	2	68% (5%)	52% (9%)	73% (6%)
SBP	control	L	250	3	68% (5%)	47% (10%)	75% (5%)
SBP	control	L	250	4	68% (5%)	47% (12%)	77% (5%)
SBP	control	L	250	5	66% (6%)	43% (9%)	79% (4%)
LDL	control	S	50	1	71% (8%)	71% (10%)	63% (5%)
LDL	control	S	50	2	68% (8%)	62% (11%)	67% (5%)
LDL	control	S	50	3	69% (8%)	66% (12%)	69% (7%)
LDL	control	S	50	4	69% (9%)	61% (15%)	69% (5%)
LDL	control	S	50	5	68% (8%)	51% (10%)	71% (5%)
LDL	control	S	100	1	70% (7%)	66% (9%)	64% (6%)
LDL	control	S	100	2	66% (9%)	55% (11%)	69% (5%)
LDL	control	S	100	3	66% (7%)	55% (15%)	70% (4%)
LDL	control	S	100	4	67% (9%)	53% (12%)	73% (7%)
LDL	control	S	100	5	65% (7%)	46% (11%)	74% (6%)
LDL	control	S	150	1	69% (8%)	63% (13%)	66% (6%)
LDL	control	S	150	2	66% (8%)	55% (14%)	70% (5%)
LDL	control	S	150	3	65% (7%)	46% (13%)	72% (5%)
LDL	control	S	150	4	65% (7%)	46% (8%)	76% (6%)
LDL	control	S	150	5	64% (8%)	36% (11%)	77% (4%)
LDL	control	S	200	1	68% (8%)	59% (12%)	66% (6%)
LDL	control	S	200	2	65% (10%)	52% (13%)	71% (5%)
LDL	control	S	200	3	64% (7%)	41% (9%)	73% (4%)
LDL	control	S	200	4	65% (6%)	42% (7%)	77% (5%)
LDL	control	S	200	5	62% (7%)	35% (12%)	78% (5%)
LDL	control	S	250	1	68% (8%)	59% (12%)	66% (6%)
LDL	control	S	250	2	64% (9%)	52% (10%)	71% (6%)
LDL	control	S	250	3	64% (6%)	42% (10%)	75% (4%)
LDL	control	S	250	4	64% (6%)	39% (8%)	78% (4%)
LDL	control	S	250	5	61% (6%)	32% (12%)	78% (4%)
LDL	control	M	50	1	71% (8%)	73% (9%)	62% (6%)
LDL	control	M	50	2	70% (9%)	67% (11%)	67% (6%)
LDL	control	M	50	3	69% (9%)	62% (13%)	69% (6%)
LDL	control	M	50	4	68% (8%)	57% (9%)	69% (6%)
LDL	control	M	50	5	69% (8%)	60% (13%)	72% (5%)
LDL	control	M	100	1	70% (8%)	68% (9%)	66% (5%)
LDL	control	M	100	2	68% (9%)	62% (9%)	69% (5%)
LDL	control	M	100	3	67% (9%)	54% (13%)	71% (6%)
LDL	control	M	100	4	65% (9%)	42% (10%)	72% (6%)
LDL	control	M	100	5	66% (6%)	49% (10%)	76% (3%)
LDL	control	M	150	1	70% (8%)	65% (9%)	66% (6%)

LDL	control	M	150	2	67% (8%)	56% (12%)	70% (5%)
LDL	control	M	150	3	66% (8%)	48% (12%)	72% (7%)
LDL	control	M	150	4	63% (8%)	37% (7%)	74% (6%)
LDL	control	M	150	5	64% (6%)	42% (10%)	77% (4%)
LDL	control	M	200	1	70% (8%)	60% (12%)	68% (5%)
LDL	control	M	200	2	66% (9%)	49% (11%)	72% (5%)
LDL	control	M	200	3	65% (8%)	46% (10%)	74% (4%)
LDL	control	M	200	4	63% (8%)	37% (8%)	76% (6%)
LDL	control	M	200	5	63% (5%)	38% (9%)	79% (3%)
LDL	control	M	250	1	69% (8%)	59% (13%)	68% (5%)
LDL	control	M	250	2	65% (8%)	49% (10%)	73% (5%)
LDL	control	M	250	3	64% (7%)	42% (9%)	74% (4%)
LDL	control	M	250	4	63% (7%)	32% (9%)	76% (6%)
LDL	control	M	250	5	62% (5%)	34% (11%)	80% (5%)
LDL	control	L	50	1	71% (7%)	72% (12%)	64% (5%)
LDL	control	L	50	2	70% (8%)	66% (13%)	67% (4%)
LDL	control	L	50	3	68% (9%)	58% (16%)	70% (7%)
LDL	control	L	50	4	70% (8%)	57% (15%)	72% (8%)
LDL	control	L	50	5	69% (8%)	51% (13%)	74% (5%)
LDL	control	L	100	1	70% (8%)	66% (12%)	66% (5%)
LDL	control	L	100	2	69% (8%)	61% (13%)	69% (6%)
LDL	control	L	100	3	67% (8%)	52% (15%)	71% (7%)
LDL	control	L	100	4	68% (8%)	47% (14%)	75% (6%)
LDL	control	L	100	5	68% (8%)	45% (11%)	76% (4%)
LDL	control	L	150	1	69% (8%)	61% (14%)	67% (5%)
LDL	control	L	150	2	68% (8%)	58% (10%)	70% (5%)
LDL	control	L	150	3	67% (7%)	47% (15%)	75% (6%)
LDL	control	L	150	4	66% (9%)	41% (16%)	77% (6%)
LDL	control	L	150	5	67% (7%)	43% (10%)	77% (5%)
LDL	control	L	200	1	69% (8%)	60% (16%)	69% (5%)
LDL	control	L	200	2	67% (8%)	55% (13%)	71% (5%)
LDL	control	L	200	3	66% (7%)	44% (13%)	76% (5%)
LDL	control	L	200	4	65% (7%)	39% (13%)	78% (5%)
LDL	control	L	200	5	65% (7%)	34% (9%)	79% (5%)
LDL	control	L	250	1	68% (8%)	58% (14%)	68% (5%)
LDL	control	L	250	2	66% (7%)	50% (10%)	71% (4%)
LDL	control	L	250	3	66% (7%)	41% (14%)	77% (6%)
LDL	control	L	250	4	64% (7%)	37% (10%)	80% (5%)
LDL	control	L	250	5	65% (7%)	35% (11%)	79% (5%)
HbA1c	improvement	S	50	1	69% (5%)	67% (8%)	59% (10%)
HbA1c	improvement	S	50	2	69% (4%)	64% (9%)	61% (8%)
HbA1c	improvement	S	50	3	68% (4%)	64% (7%)	60% (6%)
HbA1c	improvement	S	50	4	67% (6%)	62% (11%)	60% (9%)
HbA1c	improvement	S	50	5	67% (5%)	66% (9%)	62% (7%)
HbA1c	improvement	S	100	1	68% (4%)	66% (7%)	61% (11%)
HbA1c	improvement	S	100	2	67% (4%)	64% (8%)	63% (7%)
HbA1c	improvement	S	100	3	67% (5%)	62% (8%)	60% (7%)

HbA1c	improvement	S	100	4	65% (6%)	62% (8%)	62% (7%)
HbA1c	improvement	S	100	5	66% (5%)	63% (9%)	61% (8%)
HbA1c	improvement	S	150	1	68% (6%)	64% (9%)	61% (10%)
HbA1c	improvement	S	150	2	67% (4%)	62% (9%)	63% (7%)
HbA1c	improvement	S	150	3	66% (5%)	60% (7%)	62% (8%)
HbA1c	improvement	S	150	4	65% (4%)	61% (7%)	61% (7%)
HbA1c	improvement	S	150	5	66% (5%)	58% (9%)	61% (8%)
HbA1c	improvement	S	200	1	68% (5%)	65% (8%)	63% (9%)
HbA1c	improvement	S	200	2	66% (4%)	61% (9%)	62% (7%)
HbA1c	improvement	S	200	3	65% (5%)	57% (6%)	60% (6%)
HbA1c	improvement	S	200	4	64% (5%)	59% (7%)	63% (7%)
HbA1c	improvement	S	200	5	64% (5%)	58% (11%)	60% (9%)
HbA1c	improvement	S	250	1	68% (5%)	64% (8%)	60% (8%)
HbA1c	improvement	S	250	2	66% (4%)	63% (9%)	62% (7%)
HbA1c	improvement	S	250	3	64% (5%)	58% (6%)	62% (7%)
HbA1c	improvement	S	250	4	63% (5%)	58% (8%)	63% (5%)
HbA1c	improvement	S	250	5	64% (6%)	57% (10%)	62% (8%)
HbA1c	improvement	M	50	1	70% (4%)	68% (8%)	63% (8%)
HbA1c	improvement	M	50	2	69% (4%)	66% (9%)	60% (8%)
HbA1c	improvement	M	50	3	69% (4%)	65% (7%)	62% (8%)
HbA1c	improvement	M	50	4	67% (4%)	62% (8%)	62% (8%)
HbA1c	improvement	M	50	5	67% (3%)	64% (8%)	63% (7%)
HbA1c	improvement	M	100	1	70% (4%)	67% (8%)	63% (9%)
HbA1c	improvement	M	100	2	68% (3%)	64% (6%)	61% (6%)
HbA1c	improvement	M	100	3	67% (4%)	64% (7%)	61% (7%)
HbA1c	improvement	M	100	4	65% (3%)	61% (6%)	61% (4%)
HbA1c	improvement	M	100	5	67% (3%)	63% (8%)	63% (5%)
HbA1c	improvement	M	150	1	70% (4%)	66% (8%)	64% (7%)
HbA1c	improvement	M	150	2	68% (3%)	62% (7%)	64% (5%)
HbA1c	improvement	M	150	3	66% (4%)	62% (8%)	62% (6%)
HbA1c	improvement	M	150	4	64% (4%)	58% (8%)	62% (7%)
HbA1c	improvement	M	150	5	66% (4%)	62% (9%)	61% (5%)
HbA1c	improvement	M	200	1	70% (3%)	65% (10%)	65% (7%)
HbA1c	improvement	M	200	2	67% (3%)	62% (7%)	62% (6%)
HbA1c	improvement	M	200	3	66% (4%)	60% (8%)	63% (7%)
HbA1c	improvement	M	200	4	65% (4%)	57% (7%)	64% (6%)
HbA1c	improvement	M	200	5	65% (3%)	59% (8%)	60% (6%)
HbA1c	improvement	M	250	1	69% (4%)	66% (10%)	63% (6%)
HbA1c	improvement	M	250	2	67% (3%)	61% (7%)	62% (6%)
HbA1c	improvement	M	250	3	65% (4%)	59% (10%)	61% (7%)
HbA1c	improvement	M	250	4	64% (4%)	55% (8%)	64% (7%)
HbA1c	improvement	M	250	5	64% (4%)	60% (8%)	62% (6%)
HbA1c	improvement	L	50	1	70% (5%)	70% (7%)	62% (7%)
HbA1c	improvement	L	50	2	70% (4%)	67% (9%)	62% (6%)
HbA1c	improvement	L	50	3	69% (5%)	65% (10%)	63% (5%)
HbA1c	improvement	L	50	4	68% (4%)	64% (9%)	63% (5%)
HbA1c	improvement	L	50	5	68% (4%)	64% (10%)	62% (5%)

HbA1c	improvement	L	100	1	71% (5%)	67% (9%)	64% (6%)
HbA1c	improvement	L	100	2	70% (4%)	62% (9%)	63% (7%)
HbA1c	improvement	L	100	3	69% (5%)	65% (11%)	62% (5%)
HbA1c	improvement	L	100	4	67% (4%)	62% (8%)	62% (6%)
HbA1c	improvement	L	100	5	67% (4%)	61% (8%)	63% (6%)
HbA1c	improvement	L	150	1	71% (5%)	67% (9%)	64% (6%)
HbA1c	improvement	L	150	2	69% (4%)	62% (8%)	64% (6%)
HbA1c	improvement	L	150	3	68% (4%)	63% (11%)	62% (8%)
HbA1c	improvement	L	150	4	67% (5%)	61% (8%)	64% (6%)
HbA1c	improvement	L	150	5	66% (4%)	62% (6%)	62% (5%)
HbA1c	improvement	L	200	1	70% (5%)	66% (11%)	64% (6%)
HbA1c	improvement	L	200	2	68% (5%)	61% (10%)	64% (5%)
HbA1c	improvement	L	200	3	67% (4%)	62% (10%)	61% (6%)
HbA1c	improvement	L	200	4	66% (5%)	62% (10%)	64% (6%)
HbA1c	improvement	L	200	5	65% (4%)	61% (9%)	60% (5%)
HbA1c	improvement	L	250	1	70% (5%)	65% (10%)	62% (6%)
HbA1c	improvement	L	250	2	67% (4%)	61% (9%)	64% (5%)
HbA1c	improvement	L	250	3	66% (4%)	62% (9%)	64% (7%)
HbA1c	improvement	L	250	4	66% (5%)	60% (10%)	63% (6%)
HbA1c	improvement	L	250	5	64% (4%)	59% (9%)	61% (6%)
SBP	improvement	S	50	1	80% (4%)	67% (8%)	82% (5%)
SBP	improvement	S	50	2	80% (3%)	66% (7%)	83% (6%)
SBP	improvement	S	50	3	80% (4%)	67% (8%)	80% (7%)
SBP	improvement	S	50	4	80% (3%)	69% (6%)	81% (6%)
SBP	improvement	S	50	5	80% (3%)	68% (6%)	79% (6%)
SBP	improvement	S	100	1	80% (3%)	67% (7%)	82% (6%)
SBP	improvement	S	100	2	80% (4%)	67% (7%)	78% (6%)
SBP	improvement	S	100	3	80% (4%)	69% (7%)	77% (5%)
SBP	improvement	S	100	4	80% (3%)	70% (7%)	78% (5%)
SBP	improvement	S	100	5	80% (3%)	68% (6%)	81% (5%)
SBP	improvement	S	150	1	80% (3%)	67% (8%)	80% (7%)
SBP	improvement	S	150	2	80% (4%)	69% (6%)	76% (5%)
SBP	improvement	S	150	3	80% (4%)	69% (8%)	77% (4%)
SBP	improvement	S	150	4	79% (3%)	69% (7%)	79% (6%)
SBP	improvement	S	150	5	79% (3%)	66% (7%)	79% (7%)
SBP	improvement	S	200	1	80% (3%)	68% (8%)	81% (7%)
SBP	improvement	S	200	2	79% (4%)	70% (7%)	75% (5%)
SBP	improvement	S	200	3	79% (4%)	68% (7%)	77% (5%)
SBP	improvement	S	200	4	79% (3%)	69% (6%)	77% (6%)
SBP	improvement	S	200	5	78% (3%)	66% (8%)	77% (6%)
SBP	improvement	S	250	1	80% (3%)	69% (6%)	79% (7%)
SBP	improvement	S	250	2	79% (4%)	68% (9%)	76% (4%)
SBP	improvement	S	250	3	78% (4%)	68% (7%)	77% (5%)
SBP	improvement	S	250	4	79% (4%)	68% (6%)	77% (6%)
SBP	improvement	S	250	5	78% (3%)	68% (7%)	77% (7%)
SBP	improvement	M	50	1	80% (3%)	66% (8%)	82% (5%)
SBP	improvement	M	50	2	79% (3%)	68% (7%)	82% (5%)

SBP	improvement	M	50	3	80% (5%)	69% (7%)	81% (8%)
SBP	improvement	M	50	4	80% (3%)	70% (6%)	81% (7%)
SBP	improvement	M	50	5	80% (3%)	69% (7%)	80% (8%)
SBP	improvement	M	100	1	80% (3%)	68% (7%)	81% (6%)
SBP	improvement	M	100	2	79% (3%)	67% (6%)	79% (8%)
SBP	improvement	M	100	3	80% (4%)	71% (7%)	79% (6%)
SBP	improvement	M	100	4	80% (3%)	70% (6%)	79% (7%)
SBP	improvement	M	100	5	80% (3%)	69% (8%)	78% (7%)
SBP	improvement	M	150	1	81% (4%)	69% (7%)	79% (7%)
SBP	improvement	M	150	2	79% (4%)	68% (8%)	77% (9%)
SBP	improvement	M	150	3	80% (3%)	71% (8%)	78% (6%)
SBP	improvement	M	150	4	79% (3%)	69% (6%)	77% (7%)
SBP	improvement	M	150	5	80% (3%)	70% (7%)	75% (6%)
SBP	improvement	M	200	1	80% (3%)	70% (7%)	78% (8%)
SBP	improvement	M	200	2	79% (4%)	69% (7%)	77% (8%)
SBP	improvement	M	200	3	79% (3%)	70% (8%)	78% (5%)
SBP	improvement	M	200	4	79% (3%)	69% (8%)	78% (6%)
SBP	improvement	M	200	5	79% (3%)	68% (8%)	76% (7%)
SBP	improvement	M	250	1	80% (3%)	70% (6%)	77% (7%)
SBP	improvement	M	250	2	79% (4%)	69% (7%)	78% (6%)
SBP	improvement	M	250	3	78% (4%)	69% (8%)	77% (6%)
SBP	improvement	M	250	4	79% (3%)	70% (7%)	77% (7%)
SBP	improvement	M	250	5	78% (3%)	68% (7%)	75% (7%)
SBP	improvement	L	50	1	80% (3%)	66% (7%)	83% (6%)
SBP	improvement	L	50	2	80% (3%)	67% (7%)	81% (5%)
SBP	improvement	L	50	3	80% (4%)	68% (8%)	81% (7%)
SBP	improvement	L	50	4	80% (3%)	69% (6%)	79% (6%)
SBP	improvement	L	50	5	80% (4%)	68% (6%)	77% (6%)
SBP	improvement	L	100	1	80% (3%)	67% (6%)	81% (5%)
SBP	improvement	L	100	2	80% (3%)	68% (6%)	78% (8%)
SBP	improvement	L	100	3	79% (3%)	69% (7%)	78% (6%)
SBP	improvement	L	100	4	80% (3%)	71% (6%)	79% (5%)
SBP	improvement	L	100	5	80% (4%)	68% (7%)	79% (6%)
SBP	improvement	L	150	1	80% (3%)	69% (7%)	79% (7%)
SBP	improvement	L	150	2	80% (3%)	68% (6%)	76% (8%)
SBP	improvement	L	150	3	79% (4%)	68% (8%)	77% (6%)
SBP	improvement	L	150	4	80% (4%)	70% (6%)	79% (4%)
SBP	improvement	L	150	5	78% (3%)	68% (7%)	77% (6%)
SBP	improvement	L	200	1	80% (3%)	68% (7%)	77% (7%)
SBP	improvement	L	200	2	80% (4%)	69% (6%)	76% (7%)
SBP	improvement	L	200	3	79% (3%)	69% (5%)	76% (5%)
SBP	improvement	L	200	4	79% (4%)	69% (4%)	78% (5%)
SBP	improvement	L	200	5	78% (4%)	68% (7%)	76% (5%)
SBP	improvement	L	250	1	80% (3%)	70% (6%)	77% (7%)
SBP	improvement	L	250	2	80% (4%)	70% (8%)	78% (5%)
SBP	improvement	L	250	3	78% (3%)	69% (5%)	76% (4%)
SBP	improvement	L	250	4	79% (4%)	69% (5%)	77% (5%)

SBP	improvement	L	250	5	78% (4%)	69% (7%)	76% (6%)
LDL	improvement	S	50	1	76% (3%)	63% (10%)	80% (5%)
LDL	improvement	S	50	2	74% (4%)	64% (9%)	78% (5%)
LDL	improvement	S	50	3	74% (3%)	62% (8%)	78% (5%)
LDL	improvement	S	50	4	76% (3%)	64% (8%)	76% (6%)
LDL	improvement	S	50	5	74% (5%)	63% (12%)	72% (6%)
LDL	improvement	S	100	1	75% (4%)	65% (10%)	78% (5%)
LDL	improvement	S	100	2	75% (4%)	64% (8%)	75% (6%)
LDL	improvement	S	100	3	74% (3%)	62% (9%)	74% (6%)
LDL	improvement	S	100	4	74% (3%)	65% (7%)	75% (7%)
LDL	improvement	S	100	5	73% (6%)	61% (11%)	72% (5%)
LDL	improvement	S	150	1	75% (4%)	65% (10%)	75% (6%)
LDL	improvement	S	150	2	74% (4%)	64% (9%)	74% (5%)
LDL	improvement	S	150	3	73% (4%)	60% (10%)	73% (6%)
LDL	improvement	S	150	4	74% (4%)	63% (7%)	74% (6%)
LDL	improvement	S	150	5	73% (5%)	62% (12%)	73% (6%)
LDL	improvement	S	200	1	75% (4%)	64% (10%)	74% (6%)
LDL	improvement	S	200	2	74% (4%)	63% (10%)	73% (6%)
LDL	improvement	S	200	3	74% (3%)	61% (11%)	74% (6%)
LDL	improvement	S	200	4	74% (4%)	62% (9%)	73% (6%)
LDL	improvement	S	200	5	72% (5%)	62% (11%)	72% (5%)
LDL	improvement	S	250	1	75% (5%)	65% (9%)	74% (6%)
LDL	improvement	S	250	2	74% (4%)	65% (10%)	73% (5%)
LDL	improvement	S	250	3	74% (3%)	60% (10%)	74% (5%)
LDL	improvement	S	250	4	74% (4%)	62% (10%)	74% (5%)
LDL	improvement	S	250	5	72% (6%)	62% (10%)	72% (7%)
LDL	improvement	M	50	1	76% (3%)	63% (10%)	80% (5%)
LDL	improvement	M	50	2	74% (5%)	63% (10%)	77% (6%)
LDL	improvement	M	50	3	75% (5%)	63% (10%)	77% (5%)
LDL	improvement	M	50	4	75% (4%)	65% (7%)	75% (5%)
LDL	improvement	M	50	5	74% (6%)	64% (12%)	75% (6%)
LDL	improvement	M	100	1	76% (4%)	64% (10%)	78% (6%)
LDL	improvement	M	100	2	75% (5%)	63% (10%)	74% (6%)
LDL	improvement	M	100	3	74% (5%)	63% (8%)	75% (5%)
LDL	improvement	M	100	4	75% (4%)	65% (8%)	74% (7%)
LDL	improvement	M	100	5	74% (7%)	63% (11%)	75% (5%)
LDL	improvement	M	150	1	75% (5%)	63% (11%)	75% (6%)
LDL	improvement	M	150	2	74% (5%)	63% (10%)	74% (4%)
LDL	improvement	M	150	3	74% (6%)	62% (8%)	72% (6%)
LDL	improvement	M	150	4	74% (5%)	64% (10%)	73% (6%)
LDL	improvement	M	150	5	73% (7%)	62% (11%)	72% (3%)
LDL	improvement	M	200	1	75% (5%)	64% (11%)	75% (6%)
LDL	improvement	M	200	2	74% (6%)	63% (10%)	73% (4%)
LDL	improvement	M	200	3	74% (6%)	61% (10%)	73% (4%)
LDL	improvement	M	200	4	74% (5%)	64% (12%)	73% (6%)
LDL	improvement	M	200	5	73% (6%)	62% (13%)	73% (4%)
LDL	improvement	M	250	1	75% (6%)	64% (10%)	75% (6%)

LDL	improvement	M	250	2	74% (6%)	63% (10%)	72% (3%)
LDL	improvement	M	250	3	73% (6%)	61% (10%)	72% (6%)
LDL	improvement	M	250	4	74% (4%)	65% (9%)	73% (8%)
LDL	improvement	M	250	5	73% (5%)	61% (14%)	73% (5%)
LDL	improvement	L	50	1	76% (3%)	62% (10%)	79% (6%)
LDL	improvement	L	50	2	74% (5%)	62% (9%)	76% (7%)
LDL	improvement	L	50	3	75% (3%)	63% (8%)	75% (5%)
LDL	improvement	L	50	4	74% (3%)	64% (8%)	76% (7%)
LDL	improvement	L	50	5	74% (6%)	62% (10%)	76% (8%)
LDL	improvement	L	100	1	76% (4%)	64% (10%)	78% (4%)
LDL	improvement	L	100	2	74% (5%)	62% (9%)	73% (6%)
LDL	improvement	L	100	3	74% (4%)	63% (9%)	72% (6%)
LDL	improvement	L	100	4	74% (4%)	64% (8%)	73% (7%)
LDL	improvement	L	100	5	74% (6%)	64% (10%)	73% (6%)
LDL	improvement	L	150	1	75% (4%)	64% (10%)	75% (4%)
LDL	improvement	L	150	2	74% (5%)	63% (9%)	73% (6%)
LDL	improvement	L	150	3	74% (4%)	62% (10%)	75% (7%)
LDL	improvement	L	150	4	74% (4%)	63% (7%)	73% (7%)
LDL	improvement	L	150	5	74% (6%)	62% (10%)	73% (8%)
LDL	improvement	L	200	1	75% (5%)	64% (9%)	74% (4%)
LDL	improvement	L	200	2	74% (6%)	63% (7%)	73% (5%)
LDL	improvement	L	200	3	74% (4%)	62% (10%)	71% (6%)
LDL	improvement	L	200	4	73% (4%)	64% (7%)	72% (6%)
LDL	improvement	L	200	5	73% (6%)	62% (11%)	73% (7%)
LDL	improvement	L	250	1	74% (5%)	63% (8%)	74% (6%)
LDL	improvement	L	250	2	74% (6%)	63% (7%)	72% (5%)
LDL	improvement	L	250	3	73% (5%)	63% (7%)	71% (6%)
LDL	improvement	L	250	4	73% (4%)	62% (9%)	71% (6%)
LDL	improvement	L	250	5	74% (6%)	62% (9%)	72% (5%)

Supplementary Table 5. Parameters and performance across CV folds for all specifications using support vector machines

Outcome	Definition	Predictors	Cost	AUC (SD)	Sensitivity (SD)	Specificity (SD)
HbA1c	control	S	0.25	67% (6%)	61% (10%)	61% (5%)
HbA1c	control	S	0.5	67% (6%)	61% (10%)	61% (5%)
HbA1c	control	S	1	67% (6%)	61% (10%)	61% (6%)
HbA1c	control	S	2	67% (6%)	61% (10%)	61% (6%)
HbA1c	control	S	4	67% (6%)	61% (10%)	61% (6%)
HbA1c	control	M	0.25	68% (5%)	60% (9%)	63% (4%)
HbA1c	control	M	0.5	68% (5%)	60% (9%)	63% (5%)
HbA1c	control	M	1	68% (5%)	60% (9%)	63% (5%)
HbA1c	control	M	2	68% (5%)	60% (9%)	64% (5%)
HbA1c	control	M	4	68% (5%)	60% (9%)	64% (5%)
HbA1c	control	L	0.25	67% (6%)	61% (10%)	62% (8%)
HbA1c	control	L	0.5	67% (6%)	60% (10%)	62% (8%)
HbA1c	control	L	1	67% (5%)	60% (10%)	62% (8%)
HbA1c	control	L	2	67% (6%)	60% (10%)	62% (8%)
HbA1c	control	L	4	67% (6%)	61% (10%)	62% (8%)
SBP	control	S	0.25	74% (4%)	64% (8%)	70% (7%)
SBP	control	S	0.5	74% (4%)	65% (9%)	70% (8%)
SBP	control	S	1	74% (5%)	65% (9%)	70% (8%)
SBP	control	S	2	74% (5%)	66% (10%)	70% (8%)
SBP	control	S	4	74% (5%)	66% (10%)	70% (8%)
SBP	control	M	0.25	75% (4%)	65% (7%)	70% (7%)
SBP	control	M	0.5	74% (4%)	65% (7%)	70% (6%)
SBP	control	M	1	74% (4%)	64% (7%)	70% (6%)
SBP	control	M	2	74% (4%)	64% (7%)	70% (6%)
SBP	control	M	4	74% (4%)	64% (7%)	70% (6%)
SBP	control	L	0.25	74% (4%)	62% (8%)	70% (5%)
SBP	control	L	0.5	74% (4%)	62% (9%)	70% (5%)
SBP	control	L	1	74% (4%)	62% (9%)	70% (5%)
SBP	control	L	2	73% (4%)	62% (8%)	70% (5%)
SBP	control	L	4	73% (4%)	62% (8%)	69% (5%)
LDL	control	S	0.25	72% (7%)	69% (10%)	68% (7%)
LDL	control	S	0.5	72% (7%)	69% (10%)	68% (6%)
LDL	control	S	1	72% (8%)	69% (9%)	68% (6%)
LDL	control	S	2	72% (8%)	69% (9%)	68% (6%)
LDL	control	S	4	72% (8%)	69% (9%)	68% (6%)
LDL	control	M	0.25	73% (7%)	70% (11%)	65% (5%)
LDL	control	M	0.5	73% (7%)	70% (8%)	65% (5%)
LDL	control	M	1	73% (7%)	69% (7%)	65% (5%)
LDL	control	M	2	73% (7%)	69% (7%)	66% (5%)
LDL	control	M	4	73% (7%)	69% (7%)	66% (5%)
LDL	control	L	0.25	72% (8%)	70% (10%)	66% (5%)
LDL	control	L	0.5	73% (8%)	69% (11%)	66% (5%)
LDL	control	L	1	72% (8%)	69% (11%)	66% (5%)
LDL	control	L	2	72% (8%)	69% (11%)	66% (5%)
LDL	control	L	4	72% (8%)	70% (11%)	67% (5%)

HbA1c	improvement	S	0.25	70% (4%)	74% (7%)	57% (8%)
HbA1c	improvement	S	0.5	69% (4%)	74% (7%)	57% (8%)
HbA1c	improvement	S	1	70% (4%)	73% (7%)	57% (8%)
HbA1c	improvement	S	2	70% (4%)	73% (7%)	58% (8%)
HbA1c	improvement	S	4	69% (4%)	73% (6%)	58% (8%)
HbA1c	improvement	M	0.25	70% (3%)	71% (7%)	59% (6%)
HbA1c	improvement	M	0.5	70% (3%)	71% (7%)	59% (6%)
HbA1c	improvement	M	1	70% (3%)	71% (7%)	59% (6%)
HbA1c	improvement	M	2	70% (3%)	70% (6%)	59% (6%)
HbA1c	improvement	M	4	70% (3%)	71% (6%)	59% (6%)
HbA1c	improvement	L	0.25	70% (2%)	69% (8%)	61% (5%)
HbA1c	improvement	L	0.5	70% (2%)	68% (8%)	61% (5%)
HbA1c	improvement	L	1	70% (2%)	68% (8%)	62% (5%)
HbA1c	improvement	L	2	70% (2%)	68% (7%)	61% (5%)
HbA1c	improvement	L	4	70% (2%)	68% (7%)	61% (5%)
SBP	improvement	S	0.25	81% (4%)	62% (7%)	84% (5%)
SBP	improvement	S	0.5	81% (4%)	63% (8%)	84% (5%)
SBP	improvement	S	1	81% (4%)	64% (7%)	84% (5%)
SBP	improvement	S	2	81% (4%)	64% (7%)	84% (5%)
SBP	improvement	S	4	81% (4%)	64% (7%)	84% (5%)
SBP	improvement	M	0.25	81% (4%)	65% (7%)	82% (5%)
SBP	improvement	M	0.5	81% (4%)	65% (7%)	82% (5%)
SBP	improvement	M	1	81% (4%)	65% (7%)	81% (4%)
SBP	improvement	M	2	81% (4%)	65% (7%)	81% (4%)
SBP	improvement	M	4	81% (4%)	65% (7%)	81% (4%)
SBP	improvement	L	0.25	80% (4%)	65% (6%)	80% (5%)
SBP	improvement	L	0.5	80% (4%)	65% (6%)	80% (5%)
SBP	improvement	L	1	80% (4%)	65% (6%)	79% (5%)
SBP	improvement	L	2	80% (3%)	65% (6%)	79% (5%)
SBP	improvement	L	4	80% (3%)	65% (6%)	79% (5%)
LDL	improvement	S	0.25	76% (4%)	65% (8%)	76% (5%)
LDL	improvement	S	0.5	76% (4%)	65% (9%)	76% (6%)
LDL	improvement	S	1	76% (4%)	65% (9%)	76% (5%)
LDL	improvement	S	2	76% (4%)	65% (9%)	76% (5%)
LDL	improvement	S	4	76% (4%)	65% (9%)	76% (5%)
LDL	improvement	M	0.25	76% (5%)	64% (8%)	76% (6%)
LDL	improvement	M	0.5	76% (5%)	64% (8%)	77% (6%)
LDL	improvement	M	1	76% (5%)	64% (8%)	77% (6%)
LDL	improvement	M	2	76% (5%)	64% (8%)	76% (6%)
LDL	improvement	M	4	75% (5%)	64% (8%)	76% (6%)
LDL	improvement	L	0.25	75% (5%)	63% (8%)	73% (6%)
LDL	improvement	L	0.5	75% (5%)	62% (8%)	73% (6%)
LDL	improvement	L	1	75% (4%)	62% (7%)	73% (6%)
LDL	improvement	L	2	75% (4%)	62% (7%)	73% (6%)
LDL	improvement	L	4	75% (5%)	62% (7%)	72% (6%)

Supplementary Note 2. Performance on the testing set

We analyzed the performance of the models on the testing set, i.e., the remaining 20% of the CARRS sample stemming from the random split of the sample for training the algorithms. The calibration curves are displayed in Supplementary Figure 2, the performance measures in Supplementary Table 6.

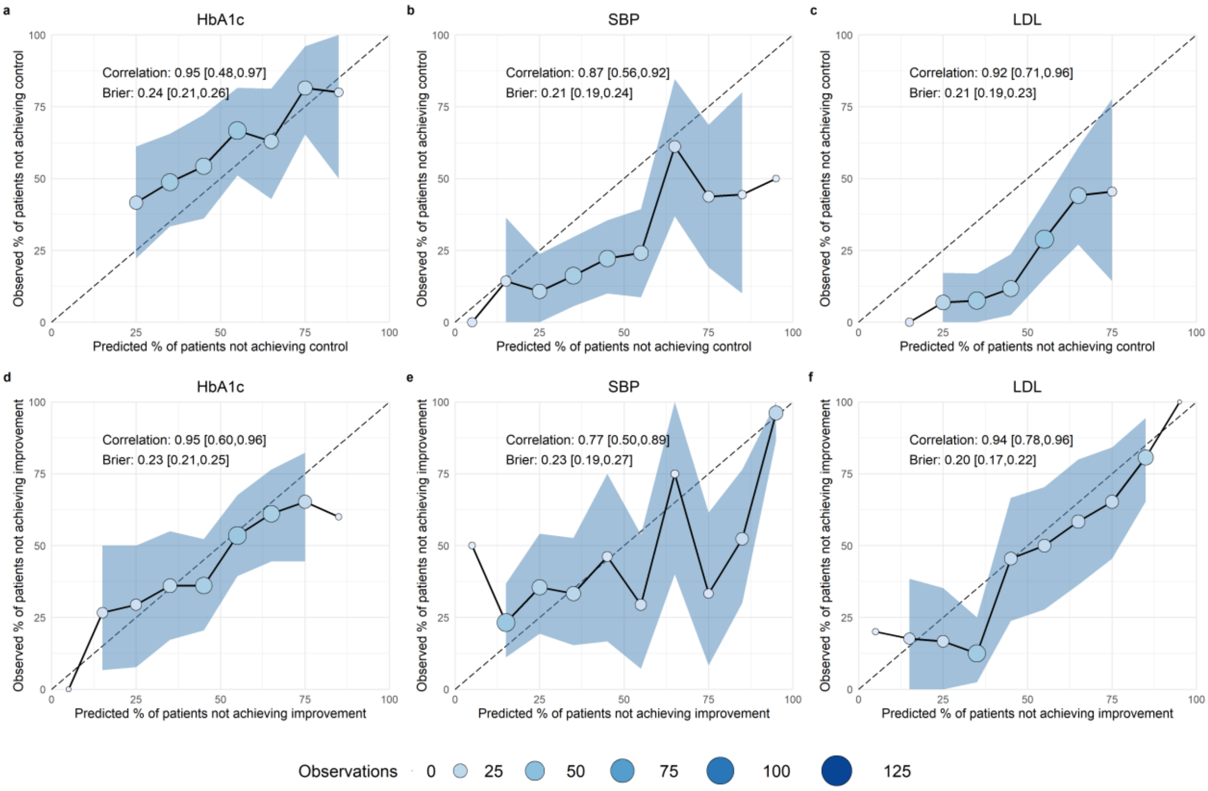

Supplementary Figure 2. Calibration curves for the testing set. Point size and fill intensity reflect the relative sample size of the respective five percentage point bin, with larger and darker points corresponding to a larger share of patients included in this bin, and empty bins not shown. a Not achieving HbA1c control. b Not achieving SBP control. c Not achieving LDL control. d Not achieving HbA1c improvement. e Not achieving SBP improvement. f Not achieving LDL improvement.

Supplementary Table 6. Performance for the testing set

Outcome definition	Not achieving control			Not achieving improvements		
**CVD risk factor**	**HbA1c**	**SBP**	**LDL**	**HbA1c**	**SBP**	**LDL**
**Chosen specification**	**Logistic (M)**	**Logistic (S)**	**Logistic (M)**	**Logistic (L)**	**Tree (S)**	**Logistic (M)**
Observations	204	204	203	203	204	204
Number of positives	123	51	42	94	90	89
Prevalence	60%	25%	21%	46%	44%	44%
[95% CI]	[53%, 67%]	[19%,31%]	[15%,26%]	[39%,53%]	[37%, 51%]	[37%, 50%]
**Targeting based on relative cutoff (top 10%)**						
Precision	85%	55%	40%	65%	100%	85%
[95% CI]	[65%,100%]	[25%,74%]	[17%,65%]	[42%,85%]	[90%,100%]	[67%,100%]
Sensitivity	14%	22%	19%	14%	22%	19%
[95% CI]	[10%, 16%]	[10%,29%]	[9%,30%]	[9%,18%]	[18%, 26%]	[14%, 23%]
**Targeting based on relative cutoff (top 10%)**						
Precision	73%	49%	42%	64%	73%	76%
[95% CI]	[61%, 86%]	[35%,63%]	[28%,56%]	[48%,76%]	[59%, 88%]	[63%, 88%]
Sensitivity	30%	49%	50%	34%	41%	44%
[95% CI]	[25%, 35%]	[38%,60%]	[35%,63%]	[26%,40%]	[34%, 50%]	[37%, 51%]
**Targeting based on relative cutoff (top 10%)**						
Precision	72%	38%	35%	59%	59%	66%
[95% CI]	[63%, 80%]	[29%,48%]	[25%,44%]	[50%,69%]	[47%, 68%]	[54%, 76%]
Sensitivity	59%	76%	83%	64%	67%	75%
[95% CI]	[54%, 65%]	[64%,86%]	[71%,93%]	[56%,71%]	[57%, 73%]	[68%, 82%]
**Targeting based on absolute cutoff**						
Detection prevalence	52%	38%	45%	53%	41%	50%
[95% CI]	[45%, 59%]	[32%,45%]	[38%,52%]	[46%,59%]	[35%, 48%]	[43%, 56%]
Precision	71%	41%	36%	59%	61%	66%
[95% CI]	[62%, 79%]	[31%,52%]	[26%,46%]	[50%,68%]	[49%, 71%]	[56%, 75%]
Sensitivity	61%	63%	79%	67%	57%	75%
[95% CI]	[52%, 69%]	[49%,77%]	[65%,91%]	[58%,77%]	[46%, 67%]	[66%, 84%]

Note: This is the performance of the models with the highest median area under the curve over all cross-validation folds applied to the testing data. Number of positives is the number of patients not achieving the outcome. Precision is the percentage of selected patients not achieving the outcome. Sensitivity is the percentage of patients not achieving the outcome who are selected. Confidence intervals obtained from bootstrapping with 1.000 draws. Sample sizes for the control and improvement definitions slightly differ due to different random sample splits, which were balanced within each outcome.

Supplementary Note 3. Sensitivity analysis based on different cutoffs

Health care providers might choose a different relative or absolute cutoff according to the capacity. For example, an increase of the cutoff would reduce the overall number of the targeted patients and improve the specificity, albeit at the costs of a lower sensitivity/reaching out to fewer patients who are at risk of missing their care target. For targeting based on ranking, the ratio of targeted and reached patients is displayed in Supplementary Figure 3.

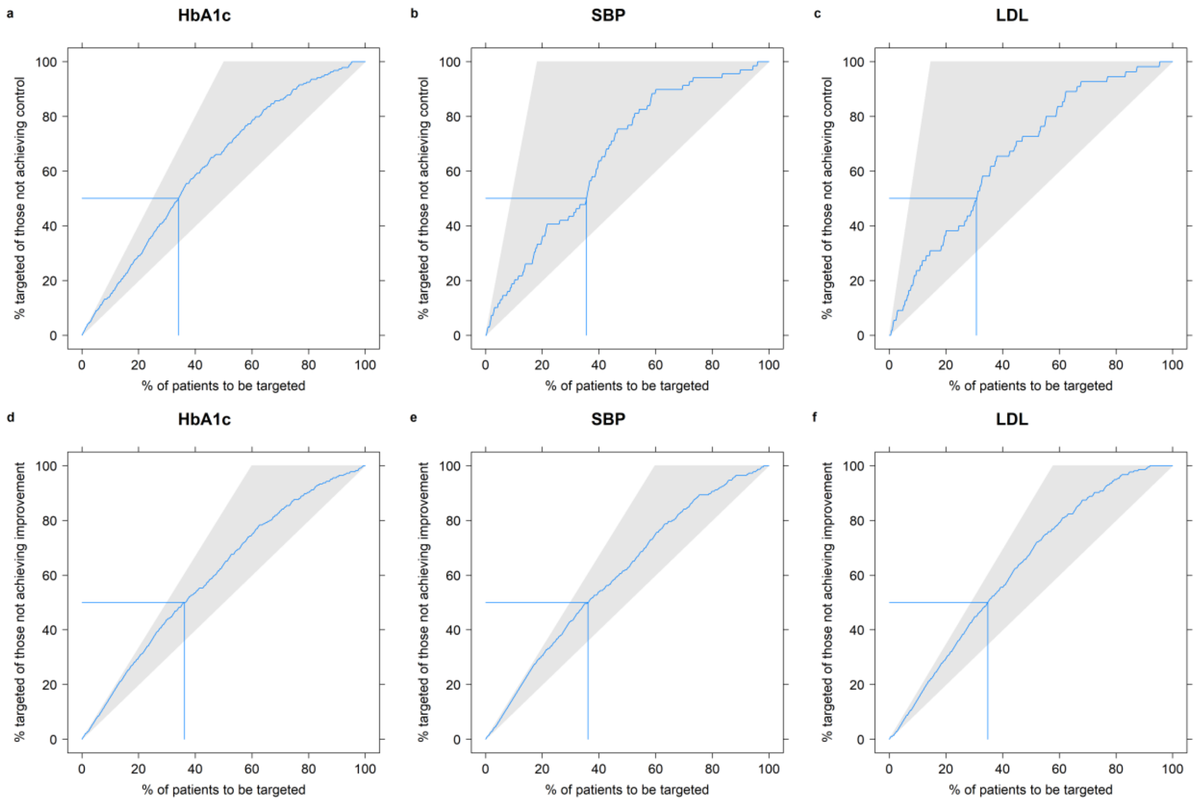

Supplementary Figure 3. Ratio of targeted and reached patients. The grey triangle represents the performance range of all possible models, with the lower border as the 45-degree line (representing the performance if a random guess was applied), and the upper two borders representing the performance of a model which could perfectly predict who is at risk. The curves represent the performance of our selected models. The straight horizontal and vertical lines mark how many of the patients not achieving CVD control respectively not achieving meaningful improvements would be sampled if the top 50% of the patients with the highest risk score were targeted. **a** Not achieving HbA1c control. **b** Not achieving SBP control. **c** Not achieving LDL control. **d** Not achieving HbA1c improvement. **e** Not achieving SBP improvement. **f** Not achieving LDL improvement.

Supplementary Note 4. Sensitivity analyses based on different timing

We assessed the performance of our models if applied on the 12-month data to predict outcomes after another 12 months (i.e., after 24 months since baseline) as a sensitivity check. The calibration curves are presented in Supplementary Figure 4, the ratio of targeted to identified patients in Supplementary Figure 5, and the performance metrics in Supplementary Table 7.

Supplementary Table 7. Performance for different timing

Outcome definition	Not achieving control			Not achieving improvements		
**CVD risk factor**	**HbA1c**	**SBP**	**LDL**	**HbA1c**	**SBP**	**LDL**
**Chosen specification**	**Logistic (M)**	**Logistic (S)**	**Logistic (M)**	**Logistic (L)**	**Tree (S)**	**Logistic (M)**
Observations	1276	1275	1277	1276	1275	1277
Number of positives	687	278	213	971	983	788
Prevalence	54%	22%	17%	76%	77%	62%
[95% CI]	[51%,57%]	[19%,24%]	[15%,19%]	[74%,79%]	[75%,80%]	[59%,64%]
**Targeting based on relative cutoff (top 10%)**						
Precision	83%	61%	50%	97%	96%	90%
[95% CI]	[76%,89%]	[52%,71%]	[42%,61%]	[93%,99%]	[93%,99%]	[84%,95%]
Sensitivity	15%	28%	30%	13%	12%	14%
[95% CI]	[14%,16%]	[24%,32%]	[25%,35%]	[12%,13%]	[12%,13%]	[13%,15%]
**Targeting based on relative cutoff (top 25%)**						
Precision	81%	47%	39%	97%	96%	87%
[95% CI]	[76%,85%]	[42%,53%]	[33%,44%]	[94%,98%]	[94%,98%]	[83%,91%]
Sensitivity	37%	54%	58%	32%	31%	35%
[95% CI]	[35%,39%]	[49%,59%]	[51%,63%]	[31%,33%]	[30%,32%]	[34%,37%]
**Targeting based on relative cutoff (top 50%)**						
Precision	74%	34%	26%	91%	91%	80%
	[70%,78%]	[31%,38%]	[23%,30%]	[89%,93%]	[88%,93%]	[76%,83%]
Sensitivity	69%	79%	79%	60%	59%	64%
[95% CI]	[66%,71%]	[75%,84%]	[72%,83%]	[58%,62%]	[57%,61%]	[62%,67%]
**Targeting based on absolute cutoff**						
Detection prevalence	28%	14%	26%	78%	80%	72%
[95% CI]	[26%,31%]	[12%,16%]	[24%,29%]	[75%,80%]	[77%,82%]	[70%,75%]
Precision	79%	56%	38%	84%	84%	72%
[95% CI]	[75%,83%]	[49%,64%]	[33%,43%]	[82%,87%]	[82%,87%]	[69%,75%]
Sensitivity	41%	36%	60%	86%	87%	84%
[95% CI]	[38%,45%]	[30%,41%]	[53%,66%]	[84%,88%]	[85%,89%]	[82%,87%]

Note: This is the performance of the models with the highest median area under the curve over all cross-validation folds applied to the time-shifted data. Detection prevalence is the percentage of patients which will be selected based on the model. Precision is the percentage of selected patients not achieving the outcome. Sensitivity is the percentage of patients not achieving the outcome who are selected. Confidence intervals obtained from bootstrapping with 1.000 draws.

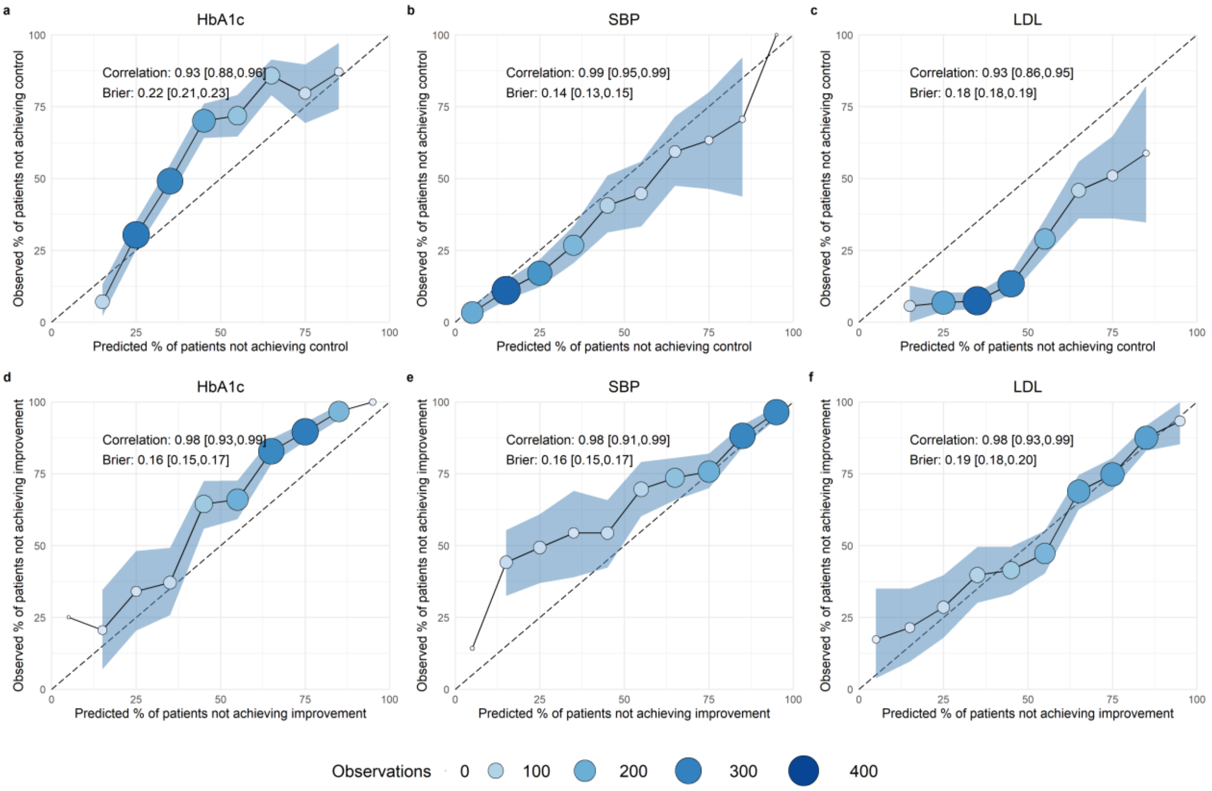

Supplementary Figure 4. Calibration curves for different timing. Point size and fill intensity reflect the relative sample size of the respective five percentage point bin, with larger and darker points corresponding to a larger share of patients included in this bin, and empty bins not shown. **a** Not achieving HbA1c control. **b** Not achieving SBP control. **c** Not achieving LDL control. **d** Not achieving HbA1c improvement. **e** Not achieving SBP improvement. **f** Not achieving LDL improvement.

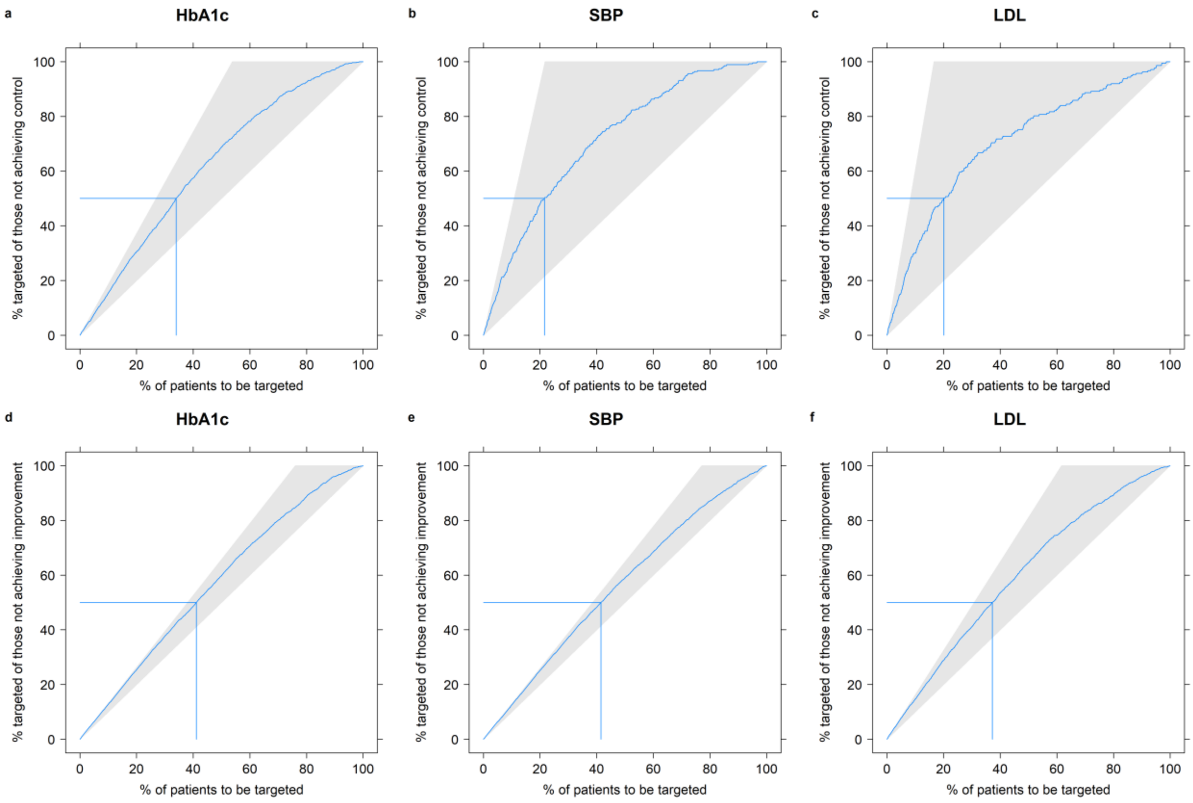

Supplementary Figure 5. Ratio of targeted patients to identified risk patients for different timing. The grey triangle represents the performance range of all possible models, with the lower border as the 45-degree line (representing the performance if a random guess was applied), and the upper two borders representing the performance of a model which could perfectly predict who is at risk. The curves represent the performance of our selected models. The straight horizontal and vertical lines mark how many of the patients not achieving CVD control respectively not achieving meaningful improvements would be sampled if the top 50% of the patients with the highest risk score were targeted. **a** Not achieving HbA1c control. **b** Not achieving SBP control. **c** Not achieving LDL control. **d** Not achieving HbA1c improvement. **e** Not achieving SBP improvement. **f** Not achieving LDL improvement.
